# Electrostatics Drive Oligomerization and Aggregation of Human Interferon Alpha-2a

**DOI:** 10.1021/acs.jpcb.1c07090

**Published:** 2021-12-13

**Authors:** Christin Pohl, Marco Polimeni, Sowmya Indrakumar, Werner Streicher, Günther H.J. Peters, Allan Nørgaard, Mikael Lund, Pernille Harris

**Affiliations:** † Novozymes A/S, Bagsvaerd, 2880, Denmark; ‡ Department of Chemistry, Technical University of Denmark, Kongens Lyngby, 2800, Denmark; § Division of Theoretical Chemistry, Department of Chemistry, Lund University, 221 00, Lund, Sweden

## Abstract

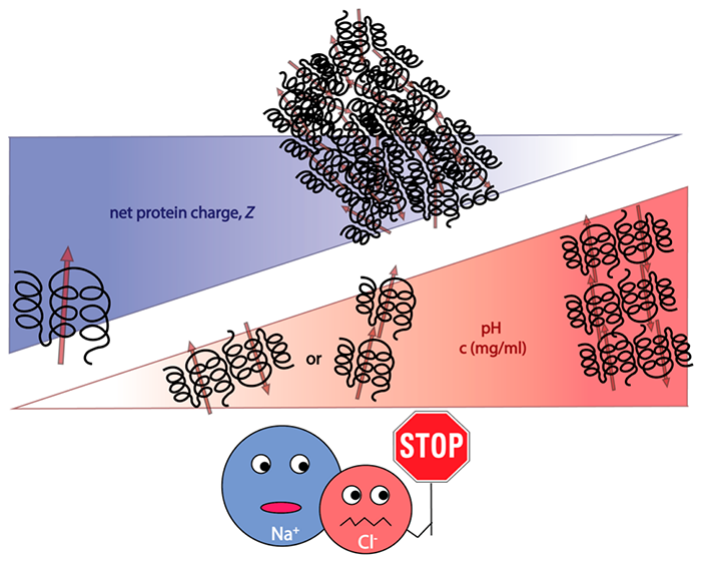

Aggregation is a common phenomenon in the field of protein therapeutics and can lead to function loss or immunogenic patient responses. Two strategies are currently used to reduce aggregation: (1) finding a suitable formulation, which is labor-intensive and requires large protein quantities, or (2) engineering the protein, which requires extensive knowledge about the protein aggregation pathway. We present a biophysical characterization of the oligomerization and aggregation processes by Interferon alpha-2a (IFNα-2a), a protein drug with antiviral and immunomodulatory properties. This study combines experimental high throughput screening with detailed investigations by small-angle X-ray scattering and analytical ultracentrifugation. Metropolis Monte Carlo simulations are used to gain insight into the underlying intermolecular interactions. IFNα-2a forms soluble oligomers that are controlled by a fast pH and concentration-dependent equilibrium. Close to the isoelectric point of 6, IFNα-2a forms insoluble aggregates which can be prevented by adding salt. We show that monomer *attraction* is driven mainly by molecular anisotropic dipole–dipole interactions that increase with increasing pH. *Repulsion* is due to monopole–monopole interactions and depends on the charge of IFNα-2a. The study highlights how combining multiple methods helps to systematically dissect the molecular mechanisms driving oligomer formation and to design ultimately efficient strategies for preventing detrimental protein aggregation.

## Introduction

The development of therapeutic protein drugs has increased dramatically in the past decade.^1,2^ Owing to their high specificity, they often show fewer side effects compared with small molecule drugs and open many possibilities for the treatment of diseases.^3^ However, they are challenging and costly in their development due to their short half-life and low stability compared to small molecules.^4−6^ In particular, the formation of oligomers and aggregates remains a challenge as they differ in their characteristics from protein to protein.^7−9^ There have been many approaches targeting this problem, but one of the challenges is the diverse behavior of different kinds of protein drugs. In this study, Interferon-alpha-2a (IFNα-2a), which is prone to aggregation^10^ is used as a model system to characterize aggregation and oligomer formation. The detailed characterization of oligomers and aggregates formed is essential to understand the mechanisms behind protein aggregation. In the long term, this knowledge can be used to alter proteins specifically to avoid oligomerization and aggregation.^11^ IFNα-2a consists of 165 amino acids (19.24 kDa) with 68% α-helical content. The five α-helices are connected through loops (Supporting Information, Figure S1A). IFNα-2a belongs to the type I interferon cytokines, which are part of the innate immune, system and is used in the treatment of, for example, hepatitis, carcinoma, leukemia, and lymphoma.^12−14^ Type I interferons show a high structural identity (Figure S1, Table S1).

IFNα was the first cytokine approved for therapeutic use by the FDA in 1986. Two IFNα-based drugs are commercially available: Roferon-A (IFNα-2a) and Intron-A1 (IFNα-2b), which only differ in one amino acid in position 23 and have similar biological activity. While Roferon-A is formulated in acetate buffer at pH 5, Intron-A is formulated in phosphate buffer at pH 7.^15^

Several studies on the stability of IFNα-2a are already published. IFNα-2a oligomerizes at pH > 5.^16^ Isothermal titration calorimetry (ITC)^17^ and stability studies using the intrinsic fluorescence of the two tryptophans in IFNα-2a^18^ have shown that temperature plays a major role in IFNα-2a’s stability. At pH 3, IFNα-2a has shown partial unfolding,^18^ while at pH 4 it seemed to be most stable.^18,20^ Aggregation and oligomerization have been observed around pH 6, which correlates with the protein’s isoelectric point (pI) of 5.98. It was shown that the addition of salt decreased IFNα-2a’s thermal stability and induced the formation of large aggregates in certain conditions.^17,20^ The addition of protein stabilizers or surfactants is a common approach to increase stability and decrease aggregation.^23,24^ For Interferon, the formulation with albumin as excipient used to be common, but a preference for albumin-free formulations due to increased aggregation^26^ led to the addition of polysorbate 80 for better storage and handling.^27^ An additional approach was the development of polyethylene glycol (PEG) conjugated IFNs, which have displayed an increased half-life.^28^ As shown from these studies, the formulation of a protein drug plays a major role in its stability. Even though many studies were performed on IFNα-2a’s stability, the correlation between protein structure, electrostatics, and aggregation propensity has, to our knowledge, not been studied so far.

IFNα-2a was selected as a representative, aggregation-prone protein to study protein self-association as a function of formulation condition. We used a combination of experimental techniques and a computational model to characterize and identify the cause of self-association. We performed high throughput screening using dynamic light scattering (DLS), isothermal chemical denaturation (ICD), and nano differential scanning fluorimetry (nanoDSF) in a pH range where IFNα-2a is known to self-associate (pH 5–9). We performed a detailed analysis and compared the oligomerization of IFNα-2a using analytical ultracentrifugation (AUC) and small-angle X-ray scattering (SAXS) on two selected pH values, below and above IFNα-2a’s pI. Additionally, we analyzed the effect of salt on oligomerization and aggregation. We performed coarse-grained Monte Carlo simulations to gain a molecular understanding of solution behavior observed experimentally. While there have been many studies to find additives or conditions to prevent aggregation, studies characterizing the aggregates formed are limited so far. This study aims to characterize the oligomers and aggregates formed by IFNα-2a and analyze the protein–protein interactions. This enables new approaches to avoid protein aggregation, such as through directed mutation of specific sites involved in protein–protein interaction or the development of PEG conjugates, which disrupt these interactions. Due to the structural similarities within this protein family (Figure S1), the results from our study may be applied to other interferons and cytokines.

## Materials and Methods

### Sample Preparation

IFNα-2a (*c* = 1.35 mg/mL) was kindly provided by Roche Diagnostics GmbH, formulated in 25 mM ammonium acetate pH 5 with 120 mM sodium chloride. If not stated otherwise, IFNα-2a samples were dialyzed into the desired condition using Slide-A-Lyzer 3500 MWCO dialysis cassettes (Thermo Fisher) as described in Pohl et al.^29^ The protein concentration after dialysis was measured using a NanoDrop 8000 spectrophotometer (Thermo Fisher). If not stated otherwise, all measurements were performed at a protein concentration of 1 mg/mL obtained by dilution into the final solution condition. Filtration of the sample and buffer was performed using Luer-Lok syringes (BD) and 0.22 μm Millex-GV filter (Merck) or 0.2 μm Whatman Anotop 10 filter (GE Healthcare Life Science) syringe filters.

### DLS Measurements

IFNα-2a samples were concentrated by centrifugation to a concentration of approximately 20 mg/mL and dialyzed in 10 mM His pH 5.5, 10 mM His pH 7, and 10 mM Tris pH 8.5 as described above. The material was filtered (0.22 μm), and the concentration was measured using a NanoDrop 8000 spectrophotometer (Thermo Fisher). A protein stock solution of 20 mg/mL was obtained by dilution with the filtered dialysis buffer (0.2 μm). The respective formulations were obtained by a 20 times dilution. The measurement was performed with a DynaPro Plate Reader II (Wyatt Technology) using Aurora 384 LV/EB plates (Brookes Life Science Systems). Silicone oil (Sigma-Aldrich) was used for sealing the wells. All measurements were performed isothermally at 25 °C with 5 s acquisition time and 20 acquisitions per well. All formulations were measured in technical triplicates. Analysis was performed using DYNAMICS version 7.8.1.3.

### NanoDSF Measurements

Sample preparation was performed likewise for DLS measurements. The measurements were performed with Prometheus NT.48 (NanoTemper Technologies). A constant temperature ramp was applied from 20 to 95 °C with 1 °C/min. The analysis was performed with PR.ThermControl (NanoTemper Technologies).

### ICD Measurements

Sample preparation was performed as described above. A protein stock solution of 1 mg/mL was prepared. Formulation buffer for the desired conditions was prepared. Denaturation buffer included 6 M GnHCl in the formulation buffer. Measurements were performed using the HUNK system (Unchained Laboratories) with no additional incubation time and a gain of 100 for fluorescence detection. The analysis was performed with Formulator (Unchained Laboratories).

### SAXS Measurements

IFNα-2a samples were concentrated to a concentration of approximately 30 mg/mL and dialyzed as described above. All samples and dialysis buffers were filtered (0.22 μm). The absorbance of all samples was measured using a NanoDrop 8000 spectrophotometer (ThermoFisher). A concentration series was obtained by dilution with the dialysis buffer. Measurements were performed at the German Electron Synchrotron DESY at the P12 EMBL BioSAXS beamline and the European Synchrotron Radiation Facility ESRF at the BM29 BioSAXS beamline. Data analysis was performed using the ATSAS software package version 2.8.4.^30^ A summary of the SAXS data collection is shown in Table S3.

### AUC Measurements

All samples were prepared as described above. The absorbance of all samples was measured using a NanoDrop 8000 spectrophotometer (ThermoFisher). All samples were diluted with dialysis buffers to an absorbance of *A*_280_ = 1, *A*_280_ = 0.5, and *A*_280_ = 0.25, measured with a NanoDrop 8000 spectrophotometer (ThermoFisher). The measurements were performed as a sedimentation velocity run with an Optima XL-1 ultracentrifuge (Beckman-Coulter) at 50000 rpm. Data analysis was performed using SEDFIT version 16.1c.^31,32^ The RMSD was used to determine the goodness of fit (see Table 1).

**Table 1:** Root Mean Square Deviation (RMSD) to Determine the Goodness of the Fit of AUC Data

	RMSD		
	*A*_280_ = 1	*A*_280_ = 0.5	*A*_280_ = 0.25
pH 5	0.0086	0.0060	0.0061
pH 5, 140 mM NaCl	0.0089	0.0060	0.0055
pH 7.5	0.0096	0.0073	0.0064
pH 7.5, 140 mM NaCl	0.0109	0.0074	0.0063
pH 7.5 concentrated	0.0115	0.0077	0.0065

Final graphs of experimental results were prepared using Origin 2019 (OriginLabs) and MATLAB (MathWorks).

### Monte Carlo Simulations

Starting from the all-atom PDB structure (PDB ID: 1ITF) a coarse-grained model of the protein, where each amino acid was replaced by a spherical bead centered in its center of mass, was constructed. Metropolis Monte Carlo simulations^33^ (MC) were performed using Faunus^34^ (v2.4.2 git revision bbd3545c) which allows for different MC moves such as protein translation and rotation, amino acid charge titration, and a cluster move (see below for more description). The potential energy function of the system was defined as
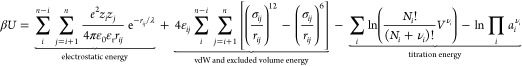


Sums run over each *i–j* amino acid pair for electrostatic, van der Waals (vdW), and excluded volume energy terms, while the titration energy was taken into account for charged amino acids. *e* is the elementary unit charge, *z*_*i*_ and *z*_*j*_ are the charges corresponding to the *i*^th^ and *j*^th^ amino acid. *r*_*ij*_ is the distance between *i* and *j*, ε_0_ is the vacuum permittivity, and *ε*_r_ = 80 is the water relative dielectric constant (i.e., the solvent is considered as a continuum homogeneous medium). Salt was included using Debye–Hückel theory through an appropriate Debye length, λ, according to the relation: λ/Å = 3.04/*I*(*M*), where *I* is the ionic strength in molar units. In the VdW term, *σ*_*ij*_ and *ε*_*ij*_ represent the minimum distance between the *i*^th^ and *j*^th^ amino acid and the energy depth which characterize their interactions. They were obtained from the Lorentz–Berthelot mixing rules,^35^*σ*_*ij*_ = (*σ*_*ii*_ + *σ*_*jj*_)/2 and , where *ii* and *jj* subscripts indicate the self-interaction parameters. The titration energy term accounts for the energy change due to the charge titration of the lateral chain of charged amino acids. For each charged amino acid species, the reaction *HA* ⇆ *H* + *A*, where *A* is a charged amino acid in its deprotonated form and *H* is a proton, is propagated back and forward using a reactive Monte Carlo scheme.^36^ To each reaction, a p*K*_a_ value is assigned based on the amino acid species (Table 2).^37^ In the equation, *N*_*i*_ is the number of amino acids of type *i*, *v*_*i*_ is the stoichiometric coefficient (positive for the products and negative for the reagents), *V* is the volume of the system, and *a*_*i*_ is the activity of the amino acid species.

**Table 2:** Reaction List for Amino Acid Titration and Relative pKa Values^37^

reaction	p*K*_a_
HCTR ⇆ H + CTR	3.67
HASP ⇆ H + ASP	3.67
HGLU ⇆ H + GLU	4.25
HHIS ⇆ H + HIS	6.54
HNTR ⇆ H + NTR	8.0
HCYS ⇆ H + CYS	8.55
HTYR ⇆ H + TYR	9.84
HLYS ⇆ H + LYS	10.4
HARG ⇆ H + ARG	12.0

**Figure 1:**
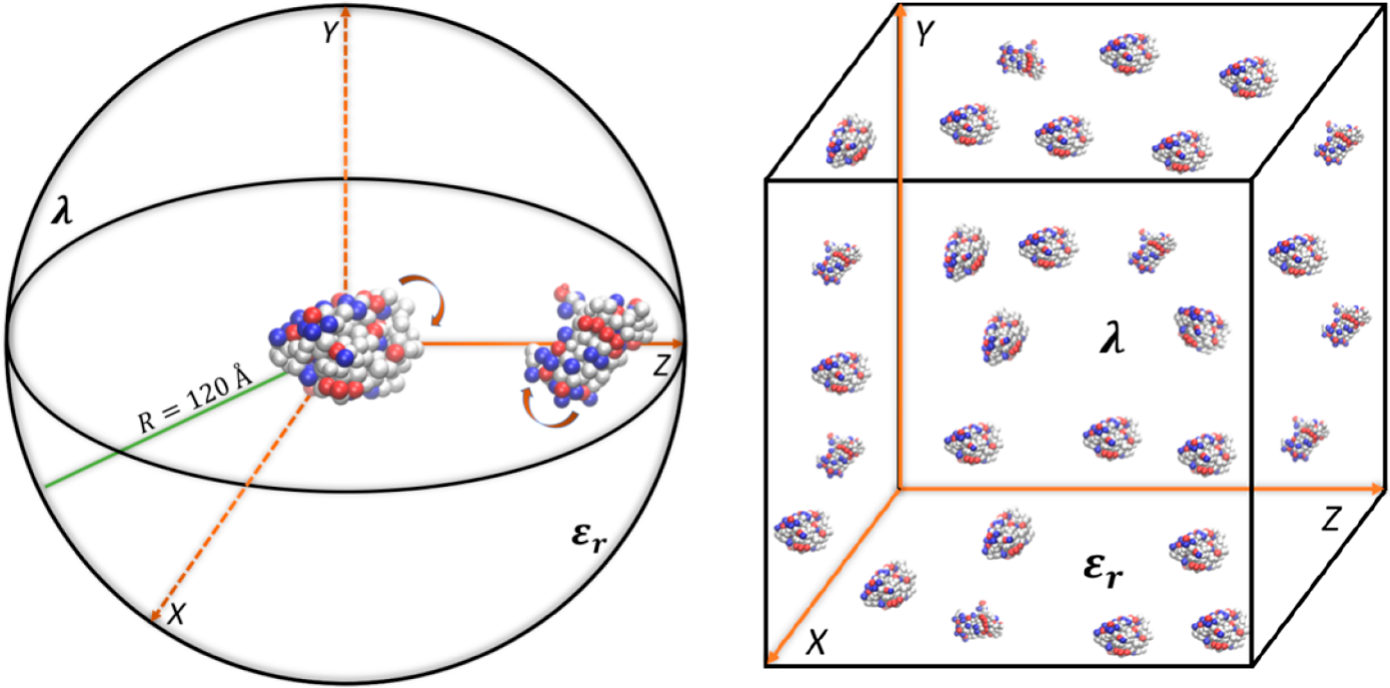
Illustration of Metropolis Monte Carlo simulations. (Left) Two-body model with one protein fixed in the center of a sphere (radius = 120 Å) which is only allowed to rotate around its center of mass. The second protein was allowed to rotate and translate along the *z*-axis. Amino acid charges could titrate according to the solution conditions (pH and ionic strength). (Right) Many-body model including 30 proteins that were allowed to translate and rotate in a periodic cube-sized to reproduce three measured protein concentrations: 1 mg/mL, 10 mg/mL, 30 mg/mL. A fixed average charge was set for each amino acid.

The solution properties were sampled using two-body and many-body models (Figure 1). A two-body model consisted of two identical proteins in a spherical cell (Figure 1 left). One protein was placed in the middle of the cell and could only rotate around its center of mass while a second one could also translate along the *z*-axis. In two-body simulations, ε_*ij*_ was set equal to 0.05 *k*_B_*T* while , where *M*_*w,i*_ is the molecular weight of the *i*^th^ amino acid and ρ = 1 g/mol/Å^3^ is an average amino acid density. For each protein–protein center of mass separation along the *z*-axis, a virtual displacement perturbation, *dL*, of the second protein was performed and the corresponding energy perturbation, *du*, was measured. The force profile, *F*(*z*), was then calculated as
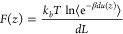
where the angle brackets, ⟨ ⟩, indicate an ensemble average. The integration of the force profile led to the potential of the mean force, *U*(*z*), which allows for the calculation of the osmotic second virial coefficient,^38^*B*_2_ as
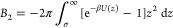
where σ is the minimum contact distance between the proteins. In this paper, we report *B*_2_ in its reduced form as
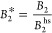
Where *B*_2_^hs^ = 2π(2*R*_h_^3^)/3 is the hard sphere contribution of a sphere of radius equal to the protein hydrodynamics radius, *R*_h_ = 22.71 Å. *B*_2_^*^ indicates a net repulsion between the proteins while a negative *B*_2_^*^ indicates a net attraction.^39^

The proteins were treated as charge distributions, and the thermally averaged electrostatic energy, *u*(z), was analyzed as a function of their mass center separation, *z*. Each protein was treated as either a monopole, a dipole, or a quadrupole, and the total multipolar interaction energy was calculated as a series of multipole terms:^40^
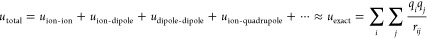
where *i* and *j* run over the charges of the first and the second protein
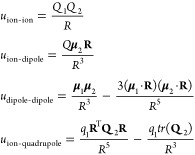
*Q*_1/2_ and **μ**_1/2_ are the total protein charges and dipole moments of the first and the second protein and . To understand what contributed more to the interaction mechanism, the ensemble average of each multipole term, *u*_*x*_, was calculated as
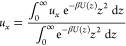
Using the two-body model, we screened a pH range from 5 to 9 (with an increment of 0.5) at 0 and 140 mM NaCl. For each solution conditions, we performed five replicas with 10^5^ equilibration steps, for charge and energy equilibration, followed by 10^6^ steps for the production runs.

**Figure 2:**
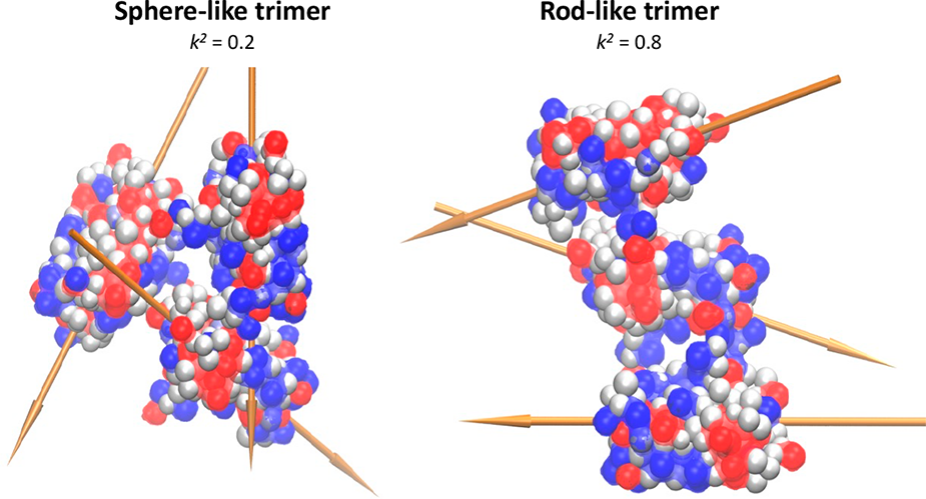
Snapshots from many-body Monte Carlo simulations showing representative structures of trimers. Left: sphere-like trimer, *k*^2^ = 0.2 (left). Right: rod-like trimer, *k*^2^ = 0.8. The golden-colored arrows represent molecular dipole moments for each of the participating proteins.

A many-body model consisted of 30 proteins in a cubic box (Figure 1 right). Each protein was allowed to translate in any direction and rotate around its center of mass. For each condition, parallel tempering was performed with 10 replicas which differed from each other by the *ε*_*ij*_ being increased by 0.01 *k*_B_*T*. The amino acid charges were equilibrated and fixed. A cluster move was performed. Two proteins were considered to start a cluster if their centers of mass were placed within a distance threshold. A third protein was considered part of the cluster if the center of mass distances between the second and the third was less than the threshold and so on. The cluster growing stopped if no further protein satisfied the threshold condition. Once the cluster was formed, it was rotated and translated as an individual object in solution using a cluster move.^41^ The cluster shape was analyzed in terms of the relative shape anisotropy,^42^*k*^2^, defined as
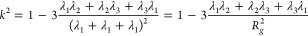
where λ_1_, λ_2_, and λ_3_ are the eigenvalues of the gyration tensor, *S*, sorted by λ_1_ ≥ λ_2_ ≥ λ_3_. *R*_*g*_ is the protein radius of gyration. *k*^2^ varies between 0 and 1, where 0 indicates a spherical symmetric cluster shape, while 1 indicates a linear chain. *k*^2^ is defined in respect to the center of mass distances, then a dimer will always have a *k*^2^ = 1. Therefore, we calculated *k*^2^ for clusters bigger than dimers. To illustrate the meaning of *k*^2^ values, the cluster shape snapshots of IFNα-2a trimers with *k*^2^ = 0.2 and *k*^2^ = 0.8 are shown in Figure 2.

## Results

### Colloidal Stability of IFNα-2a

**Figure 3:**
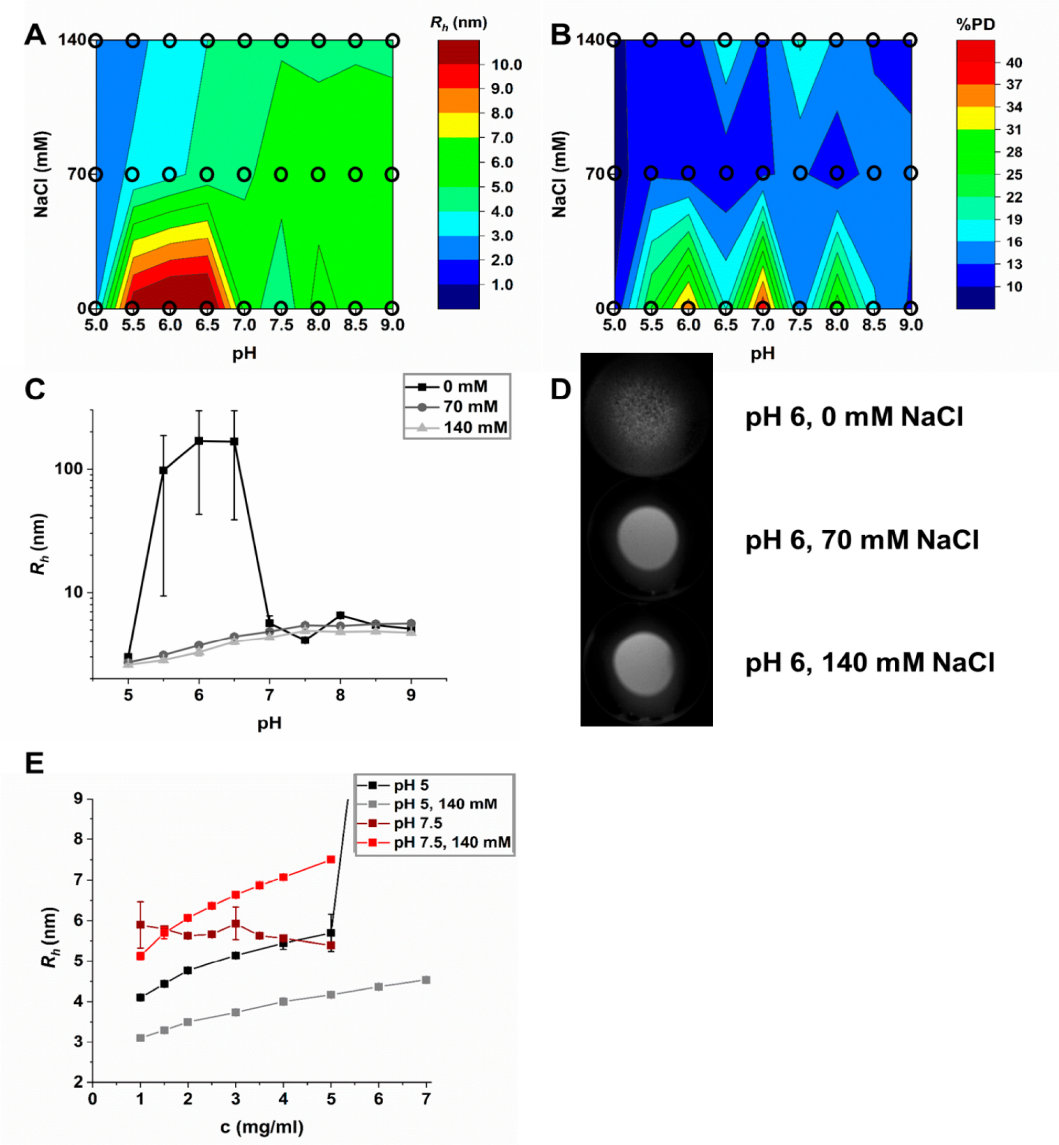
(A) Contour plot of the hydrodynamic radius *R*_h_ (nm) of INFα-2a measured with DLS as a function of pH and salt (NaCl) concentration. (B) %Polydispersity. Formulations measured are indicated as dots. (C) Line plots of *R*_h_ measurements. Data are mean ± SD for 3 replicates. (D) Visual inspection of IFNα-2a sample taken at pH 6 with 0, 70, and 140 mM NaCl. (E) *R*_h_ of INFα-2a at pH 5 and pH 7.5 in the presence and absence of salt at various protein concentrations (see also Figure S4). Measured conditions are indicated by black circles.

The colloidal stability of IFNα-2a was investigated using DLS (Figure 3) at different solution conditions (pH, added salt, and protein concentration). With no salt, the measurements showed a dramatic increase and high standard deviation of the derived hydrodynamic radius *R*_h_ around pH 6, indicating the formation of large aggregates (Figure 3A,C). These aggregates were also observed upon visual inspection (Figure 3D). With salt (NaCl), we observed no visible aggregation. The polydispersity within the sample showed lower variation in the presence of salt (Figure 3B) resulting in more homogeneous results for *R*_h_ (Figure 3C). This indicated higher colloidal stability in the presence of salt. Over the whole pH range, IFNα-2a showed an increasing *R*_h_ upon increasing pH (Figure 3C), indicating an increased propensity to form reversible soluble oligomers. Under all measured conditions, the derived *R*_h_ was higher than the theoretical *R*_h_ of IFNα-2a of 2.27, calculated from the monomeric structure^43^ (Table S4). We observed no stabilizing effect of different buffer systems (acetate and phosphate) or by the addition of excipients (280 mM sucrose, 140 mM arginine, 280 mM proline) (Figure S3). Concentration by centrifugation and subsequent dilution had no effect on *R*_h_.

This high throughput screening was used to identify conditions to further investigate the colloidal stability in detail at pH 5 and pH 7.5 (below and above pI) with and without salt. We investigated IFNα-2a at various protein concentrations using DLS (Figure 3E). At pH 5 the *R*_h_ of IFNα-2a showed an increasing trend toward higher protein concentration indicating the formation of soluble oligomers. At pH 7.5 in the presence of salt, we observed a similar increasing trend. Without salt, *R*_h_ did not increase at higher protein concentration but showed higher polydispersity and standard deviation (see also Figure S4). This indicates the presence of a small fraction of insoluble aggregates. Generally, *R*_h_ was lower when salt was present. At higher protein concentrations large insoluble aggregates formed in the absence of salt (see also Figure S4).

### Structural Analysis of IFNα-2a Using SAXS

**Figure 4:**
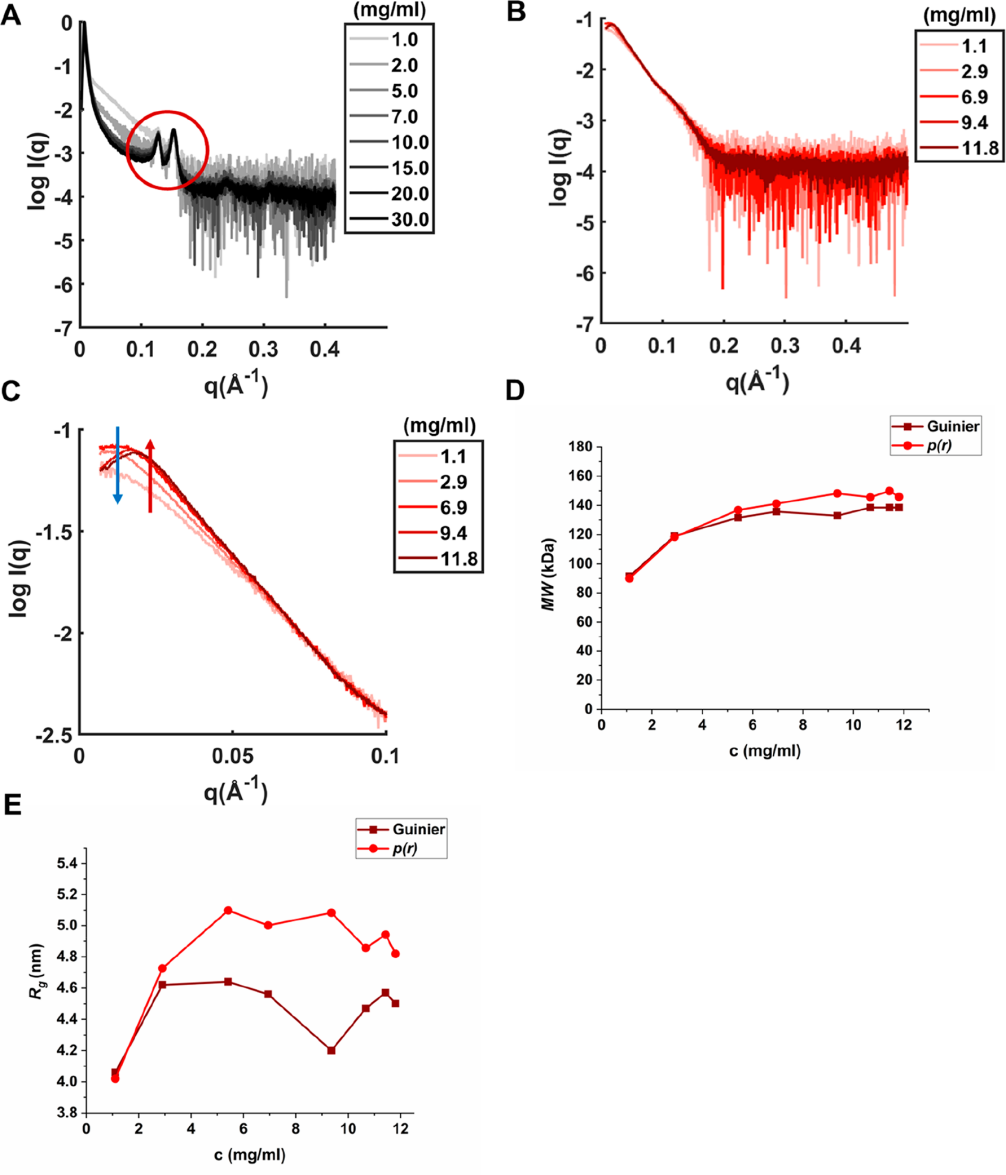
SAXS measurements of IFNα-2a in histidine at pH 5 and pH 7.5. (A) SAXS curves in histidine pH 5. Bragg peaks are indicated by a red circle. (B) SAXS scattering curves in histidine pH 7.5. (C) Scattering intensities at low *q*(Å^–1^) at pH 7.5. Oligomerization or aggregation is indicated by the red arrow; repulsion is indicated by the blue arrow. (D) Derived apparent molecular weight MW from Guinier analysis and *p*(*r*) analysis at pH 7.5. (E) Derived radius of gyration *R*_g_ from Guinier analysis (dark red) and *p*(*r*) analysis (red) at pH 7.5. For distance distribution functions, *p*(*r*), see Figure S6.

We measured SAXS at pH 5 and pH 7.5 to investigate the colloidal stability further and structurally analyze the oligomers formed by IFNα-2a. With no salt present, the SAXS measurements of IFNα-2a at pH 5 showed Bragg peaks in the SAXS curve (Figure 4A), indicating the formation of protein crystals, which were confirmed upon visual inspection under a microscope. The protein crystal formation appeared to be independent of the buffer system (Figure S5). At pH 7.5 the derived apparent molecular weight (MW) and radius of gyration (*R*_g_) increased with protein concentration (Figure 4). This was contradictory to the DLS measurements but might be explained by the high sensitivity of DLS toward aggregates present in the sample, introducing higher standard deviation and polydispersity.^44^ Our SAXS measurements revealed the simultaneous presence of different-sized soluble oligomers, which is in agreement with previous studies on IFNα-2b.^45^ The derived *R*_g_ showed a deviation between Guinier analysis and *p*(*r*), most likely due to present insoluble aggregates, which have a larger impact on the analysis when the Guinier approximation is used. The upper part of the SAXS curve (low *q*(Å^–1^)), which indicates protein–protein interactions, showed increased scattering intensity (red arrow) but also a decrease in scattering intensity at higher protein concentrations (blue arrows) (Figure 4C). This might be explained by increased self-association propensity at higher protein concentration, but repulsion is still present in the sample. A change in the buffer system did not change the overall shape of the SAXS curve of IFNα-2a at pH 7.5 (Figure S5).

**Figure 5:**
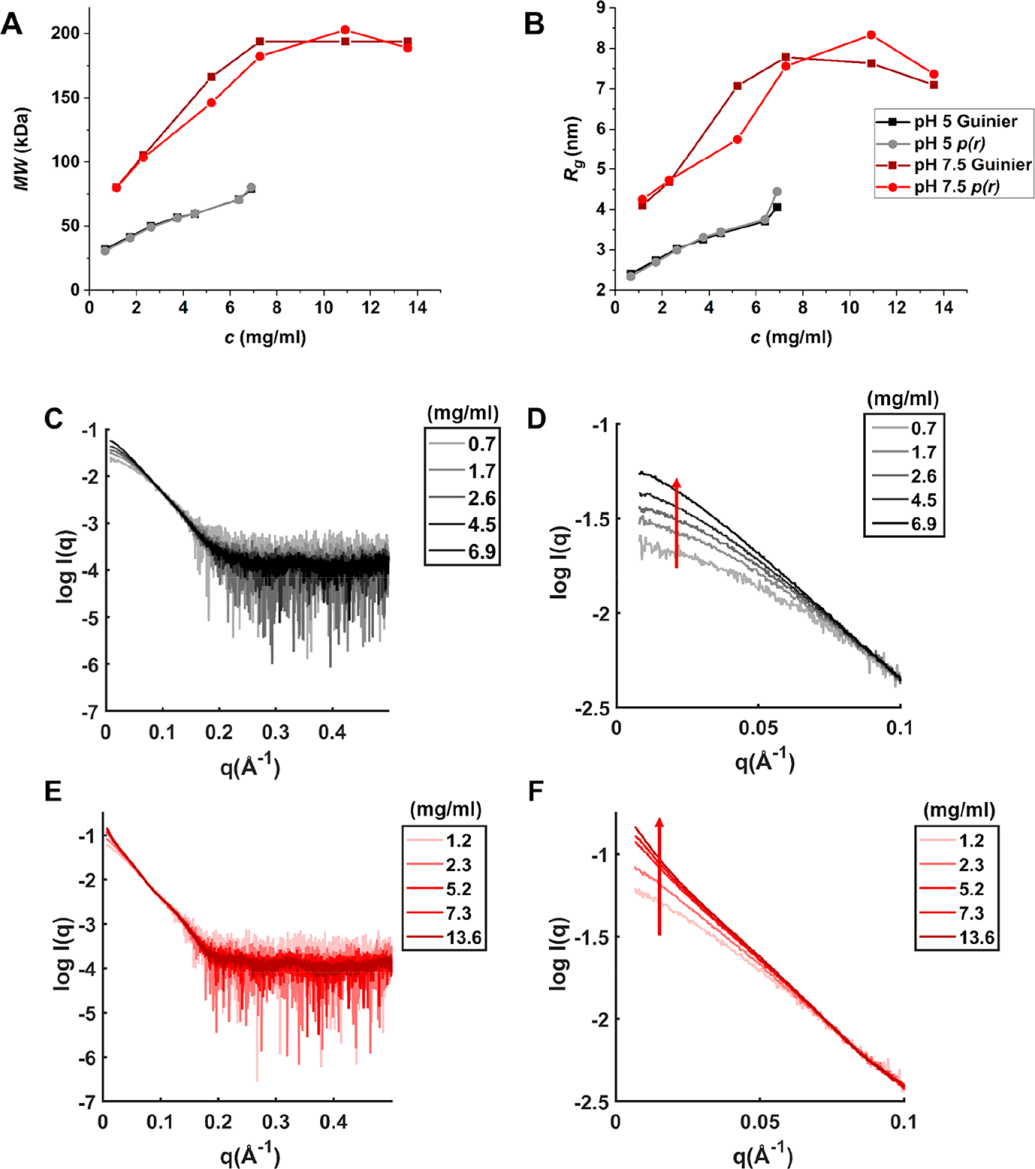
SAXS measurements of IFNα-2a in histidine at pH 5 and pH 7.5 with 140 mM NaCl. (A) Derived apparent MW from Guinier analysis and *p*(*r*) analysis. (B) Derived radius of gyration *R*_g_ from Guinier analysis and *p*(*r*) analysis. (C) SAXS scattering curves at pH 5. (D) Scattering intensities at low *q*(Å^–1^) at pH 5. Oligomerization/aggregation is indicated by the red arrow. (E) SAXS scattering curves at pH 7.5. (F) Scattering intensities at low *q*(Å^–1^) at pH 7.5. Oligomerization/aggregation is indicated by the red arrow.

In the presence of salt (140 mM NaCl), no IFNα-2a protein crystals formed. IFNα-2a showed a significantly lower apparent MW and smaller *R*_g_ at pH 5 than at pH 7.5 (Figure 5A,B), which agrees with the results from DLS. Similar to the measurements in the absence of salt, we observed an increase of apparent MW and *R*_g_ with increasing protein concentration. The scattering intensities at low *q*(Å^–1^) indicated increased self-association at higher protein concentrations (Figure 5D,F). This was more pronounced at pH 7.5. With added salt, we observed a reduction of repulsion, but the overall shape of the SAXS scattering curves was unaffected by the presence of salt (Figure 5F, Figure S7), indicating the formation of similar soluble oligomers.

To characterize the structure of the oligomer formed, we attempted to use *ab initio* modeling using DAMMIF^46^ and GASBOR.^47^*Ab initio* modeling determines the average particle in solution. We observed an elongated screw-shaped form as a common feature for the oligomers (Figure S8), but due to the simultaneous presence of different sized oligomers the exact assembly of IFNα-2a could not be modeled (see also supplementary discussion).

### Oligomeric Species of IFNα-2a Analyzed by AUC

**Figure 6:**
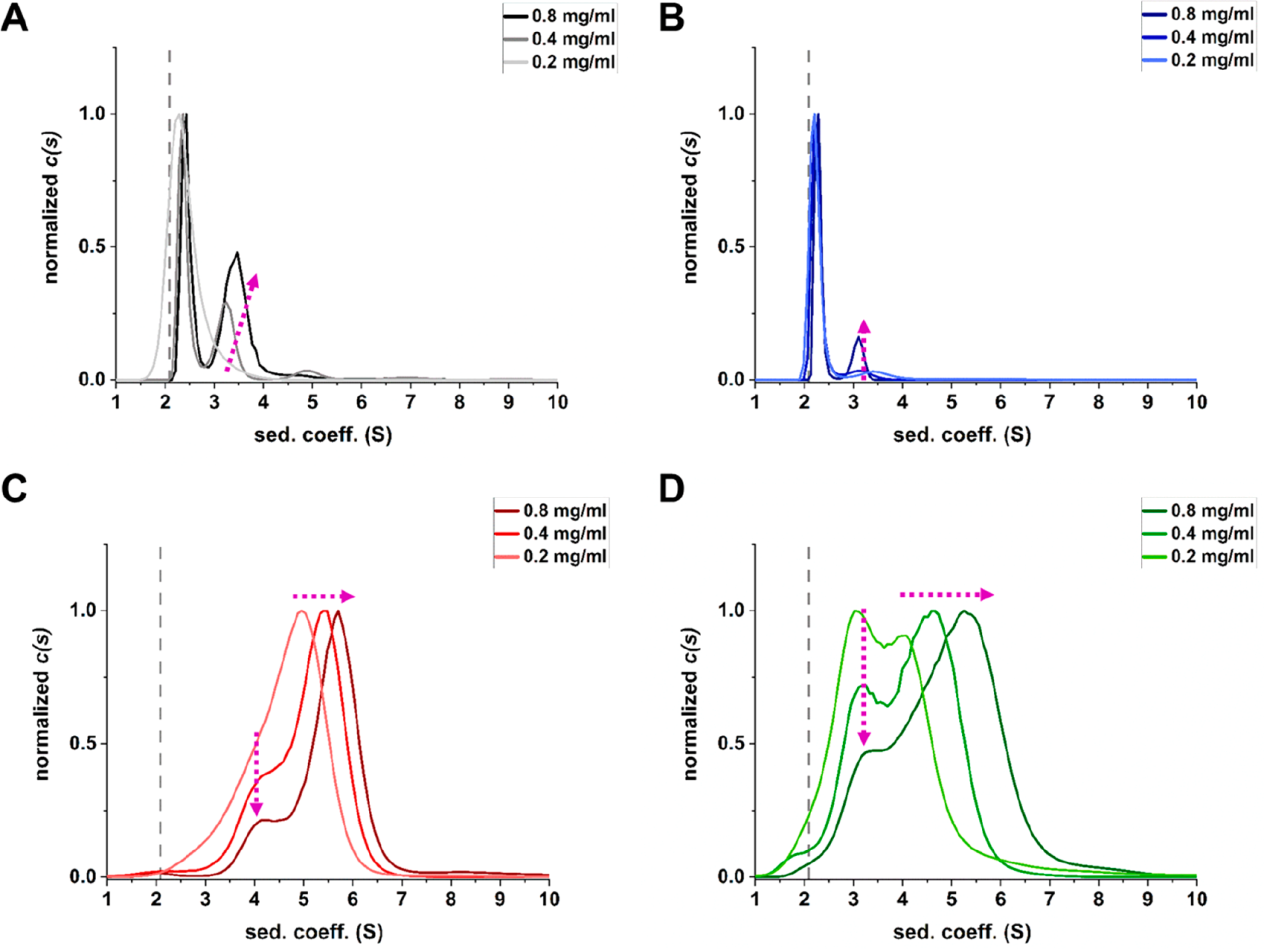
Analysis of IFNα-2a oligomers present in different conditions measured with AUC. (A) histidine pH 5, 0 mM NaCl. (B) histidine pH 5, 140 mM NaCl. (C) histidine pH 7.5, 0 mM NaCl. (D) histidine pH 7.5, 140 mM NaCl. All measurements were performed at *c* = 0.8 mg/mL (*A*_280_ = 1), *c* = 0.4 mg/mL (*A*_280_ = 0.5) and *c* = 0.2 mg/mL (*A*_280_ = 0.25) and analyzed with sedfit 16.1c.^32^ The dashed line indicates the theoretical sedimentation coefficient of the monomer (*s* = 2.08) based on alignment to the crystal structure of IFNβ (PDB:1AU1)^50^ calculated with HULLRAD.^43^

To examine which oligomeric species of IFNα-2a were present, we performed AUC. IFNα-2a was measured in histidine buffer pH 5 and pH 7.5 at different protein concentrations (*c* = 0.8 mg/mL (*A*_280_ = 1), *c* = 0.4 mg/mL (*A*_280_ = 0.5) *c* = 0.2 mg/mL (*A*_280_ = 0.25)) in the presence or absence of salt (Figure 6). We calculated the sedimentation coefficient from monomeric IFNα-2a to *s* = 2.08^43^ (dashed line). Soluble oligomeric species were present in all conditions but to different extents (Table S5). At pH 5, IFNα-2a was mainly monomeric. A second oligomeric species was present at pH 5, which showed a concentration-dependent increase. This was more pronounced in the salt-free case. Remarkably, with higher protein concentration this fraction showed a higher sedimentation coefficient, indicating a fast equilibrium between different soluble oligomeric species. This equilibrium shifts toward larger species at higher protein concentrations, which agreed with the SAXS measurements. At pH 5, this fraction corresponded to a dimer in equilibrium with higher oligomeric species. At pH 7.5, IFNα-2a was mainly present as soluble oligomeric species of different sizes and only a small fraction was monomeric. Two fractions of different oligomeric species were separated. The smaller oligomeric species corresponded most likely to dimers, and its proportion decreased at higher protein concentration. The proportion of larger oligomers increased with concentration and showed a concentration-dependent equilibrium toward larger oligomeric species. The proportion of larger oligomers appeared to be higher without salt than with salt. A comparison of different formulated samples indicated no significant difference in oligomers present in the same condition (Figure S9). When no salt was present, a small fraction of very large species corresponding to insoluble aggregates were present, which is in agreement with our DLS measurements.

### Metropolis Monte Carlo Simulations

**Figure 7:**
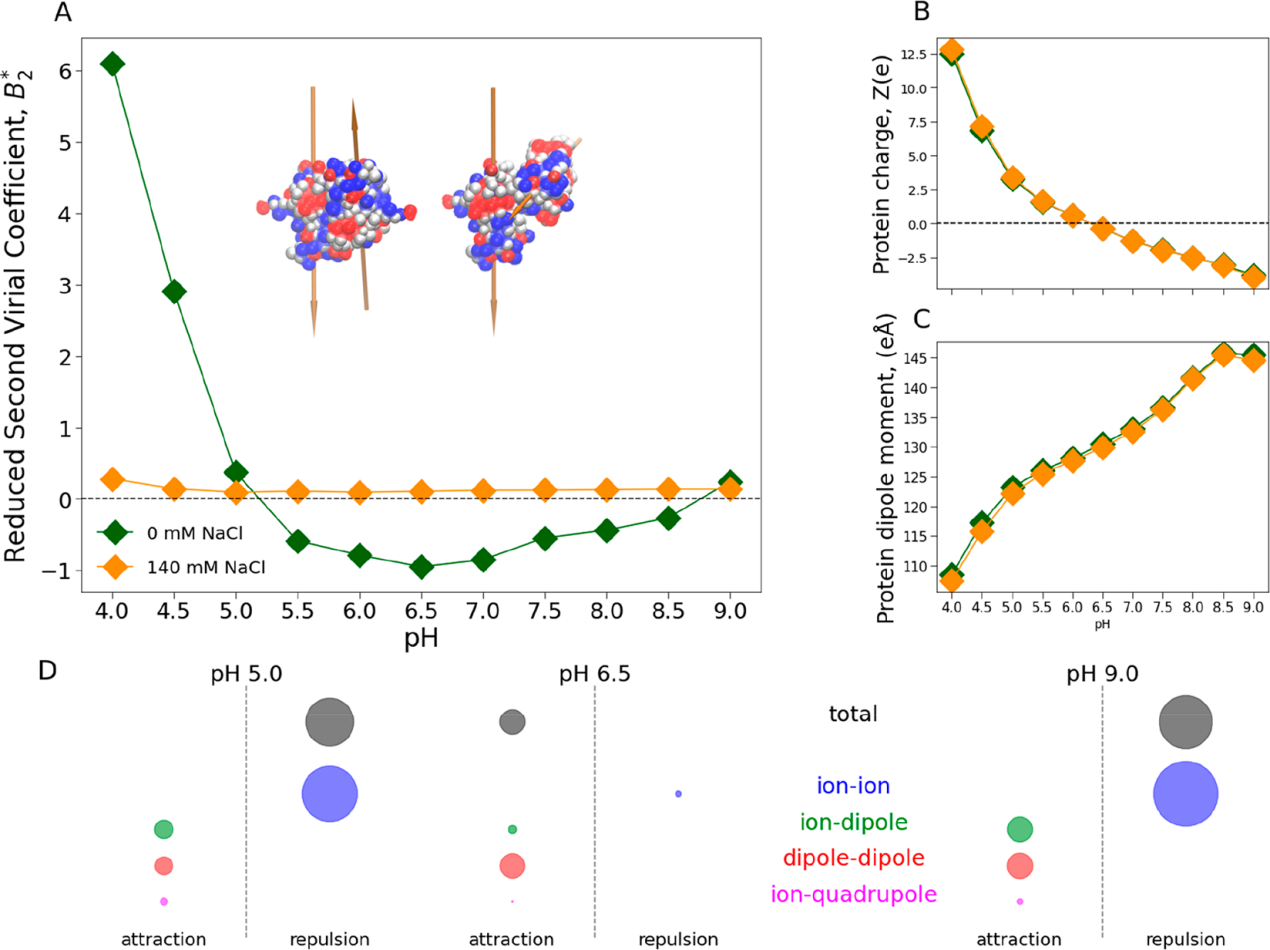
Two-body coarse-grained Metropolis Monte Carlo simulations as a function of pH with 0 mM (green) and 140 mM NaCl (orange). (A) Reduced second osmotic virial coefficient, *B*_2_^*^, and snapshots of the dipole orientations corresponding to the minimum energy configurations at pH = pI. The arrows, pointing from the negative to the positive charge, represent the dipole magnitudes (≈122 *e*Å or ≈25 Debye). (B) Total protein charge, *Z*. (C) Net protein dipole moment, μ. (D) Electric multipole contributions to the protein–protein interaction for pH 5, 6.5, and 9 (0 mM NaCl). The circle area illustrates the magnitude of the contribution. Negative (attractive) and positive (repulsive) contributions are separated by the dashed line. The sum of all multipole contributions is represented by the gray circle (“total”).

To obtain insight into the molecular mechanism driving the aggregation/oligomer formation of the IFNα-2a solution, we applied a combination of Metropolis Monte Carlo simulations and coarse-grained modeling. Details regarding the simulations and models used are described in the Materials and Methods section. Figure 7 shows the calculated reduced second osmotic virial coefficient, *B*_2_^*^ = *B*_2_/*B*_2_^hs^, the net protein charge, *Z*, and the protein dipole, μ, as a function of pH, with no added salt and with 140 mM of NaCl. The simulations were performed using a two-body model, that is, where only two proteins were embedded in the aqueous salt solution.

Our simulations showed that without salt, *B*_2_^*^ is strongly pH-dependent. At low and high pH IFNα-2a showed net repulsion in solution, indicated by positive *B*_2_^*^. At neutral pH, *B*_2_^*^ was negative indicating attraction, with the highest attraction at pH 6.5, corresponding approximately to the protein’s pI. With added salt, IFNα-2a showed constant net repulsion in solution independent of pH. These results were in very good agreement with the colloidal stability of IFNα-2a measured with DLS, indicating that our model captured the main molecular mechanisms leading to self-association of IFNα-2a.

Treating protein charge distribution as a set of electrostatic multipoles, we first investigated the protein net charge, *Z*, and the protein dipole, μ (Figure 7B,C). As expected, the absolute value of *Z* was high at pH far from the protein’s pI (pH ≈ 6) and zero at the pI. μ showed pH dependence and increases with pH. We analyzed the multipole terms in the absence of salt to investigate their contribution to protein–protein interaction of IFNα-2a (Figure 7D). We saw that repulsion was driven by ion–ion contributions. The attraction was driven by ion–dipole, dipole–dipole, and ion–quadrupole contributions. At low and high pH, where IFNα-2a was highly charged, ion–ion interactions dominated the system, leading to overall repulsion. Around the protein’s isoelectric point with little or no protein charge, dipole–dipole contributions dominated, leading to overall attraction. With added salt, electrostatic contributions were effectively screened, independent of pH. This electrostatic behavior agreed with our SAXS measurements where we observed a reduction in repulsion when adding salt. Using the graphical software VMD,^51^ we analyzed the dipole–dipole orientation during the simulations. The energy configurations were minimized at antiparallel or head-to-tail orientation (Figure 7A), indicating these as preferred orientations.

To investigate the formation of clusters (or soluble oligomers), we used a many-body model where multiple proteins are allowed to interact. We analyzed the average cluster size and shape as a function of pH with and without salt. The strength of the Lennard-Jones interactions, represented by the *ε*_*ij*_ parameter, was also explored using the parallel tempering method (see Materials and Method section). Increasing *ε*_*ij*_ can be approximately interpreted as a lowering of the system temperature.

**Figure 8:**
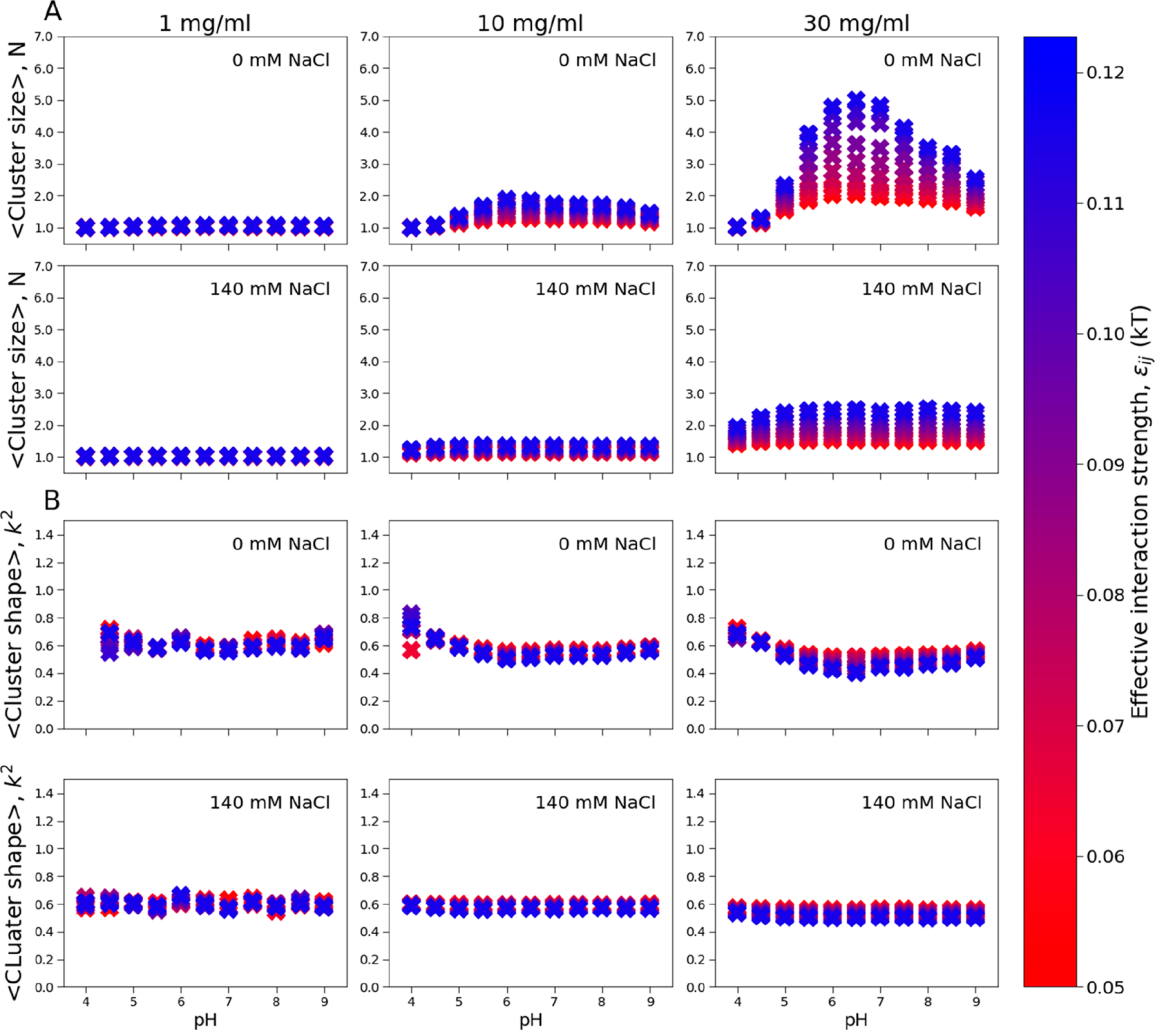
Cluster formation of IFNα-2a in many-body coarse-grained Metropolis Monte Carlo simulations. (A) Average cluster size. *N* indicates the average number of monomers in the cluster observed during the simulations. (B) Average cluster shape. *k*^2^ is the relative shape anisotropy. *k*^2^ close to 0 indicates a spherical cluster while a *k*^2^ close to 1 indicates an elongated, rod-like structure. By definition, *k*^2^ is equal to 1 for dimers, so the analysis is reported only for trimers or larger. Clustering is shown as a function of pH at three protein concentrations (1, 10, and 30 mg/mL), and at 0 mM and 140 mM NaCl. For each condition replica exchange simulations are reported for *ε*_*ij*_ values in the range of 0.05 kT (red) to 0.012 *k*_B_*T* (blue).

At low protein concentration, the average cluster size is close to 1, meaning that only monomers were observed (Figure 8A, left). At higher protein concentration, the average cluster size increases with increasing pH (Figure 8A, right). The largest clusters are formed around the protein’s pI and at a lower temperature (high *ε*_*ij*_). This effect was less pronounced in the presence of salt, due to screening of the electrostatics. These results correlate very well with our experimental observations where we found increased oligomerization with increasing pH and a pronounced oligomer formation around the protein pI.

We additionally analyzed the average cluster shape by determining the shape anisotropy parameter, *k*^2^, a method that is commonly applied to polymers.^52^ We observed more spherical cluster shapes (*k*^*2*^) with increasing cluster size, indicating a less defined orientation for larger oligomers (Figure 8B). Snapshots of IFNα-2a trimers, corresponding to different *k*^2^ values, are shown in the Materials and Methods section.

### Stability toward Thermal and Chemical Denaturation of IFNα-2a

**Figure 9:**
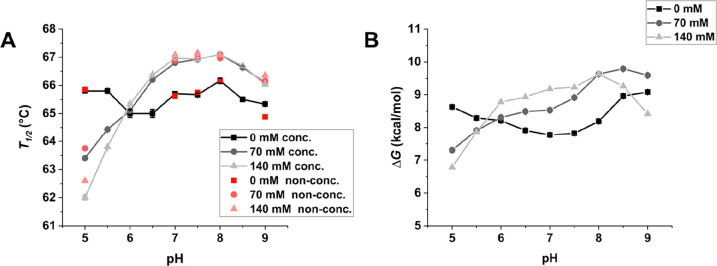
(A) Thermal denaturation point *T*_1/2_ of INFα-2a measured with nanoDSF at different formulation procedures. A sample concentrated before dialysis (conc., black) and a nonconcentrated sample (non-conc., red) were measured. Data are mean ±SD for three replicates (error bars obscured by points). (B) Stability of INFα-2a toward chemical denaturation measured with ICD in the presence of 0, 70, and 140 mM NaCl as a function of pH.

In addition to the colloidal stability of IFNα-2a, we studied the conformational stability as a function of pH at 0, 70, and 140 mM NaCl. The thermally induced unfolding (*T*_1/2_) of IFNα-2a was measured using nanoDSF (Figure 9A). With no salt present, IFNα-2a unfolded at around 65 °C, independent of pH. With added salt (70 and 140 mM NaCl), *T*_1/2_ of IFNα-2a was highly pH-dependent. NaCl showed a destabilizing effect on the thermal stability below pH 6 and a stabilizing effect above pH 6. We tested the influence of different formulation procedures of IFNα-2a. *T*_1/2_ of the sample concentrated before dialysis (black and gray) was in good agreement with the nonconcentrated sample (red). We determined the free energy changes by denaturant-induced unfolding (Δ*G*) with ICD (Figure 9B). The conformational stability toward chemically induced denaturation showed a similar trend as seen for thermal-induced denaturation. Remarkably, the curves for both *T*_1/2_ and Δ*G* cross at pH 6, correlating with the protein’s pI of 5.98. The conformational stability of IFNα-2a was additionally investigated in different buffer systems (acetate pH 5 and phosphate pH 7.5) and in addition of selected excipients (sucrose (280 mM), arginine·HCl (140 mM) and proline (280 mM), but no significant changes could be seen (Figure S11).

## Discussion

IFNα-2a is widely used as a therapeutic drug against various diseases^12−14^ but has shown difficulties in formulation due to aggregation. We assessed the colloidal stability of IFNα-2a as a function of pH experimentally and through molecular simulations. Our experimental results were in excellent agreement with our simulations, indicating that the main factors leading to IFNα-2a aggregation were captured by the simulation models. We observed increased formation of soluble oligomers with increasing pH and the formation of large insoluble aggregates around the protein’s pI of 5.98 experimentally as well as in our simulations, which is in agreement with previous studies.^16^ The addition of salt prevented the formation of insoluble aggregates and shifted the equilibrium of soluble oligomers slightly toward smaller species. The effect of salt on protein stability is poorly understood, but molecules with significant dipole moment were shown to be stabilized by the addition of salt through screening of the anisotropic charge distribution within the protein.^57,58^ With added salt, a positive *B*_2_ indicated repulsion independent of pH, thus supporting the idea that nonspecific ion binding and effective screening of charges stabilized IFNα-2a. In our simulations, the only electrostatic contribution for repulsive behavior was pH dependent due to ion–ion interactions. Attraction instead came mainly from dipole–dipole interactions. Further, the dipole moment of IFNα-2a showed a pH-dependent increase, which might explain increased formation of soluble oligomers seen in our experiments. Huang et al. showed that the addition of salt led to increased attraction.^17^ However, in our study, we showed that *B*_2_ is highly pH-dependent in the absence of salt, and salt-induced attraction thus cannot be generalized.

Our experimental results revealed the simultaneous presence of different species of soluble oligomers that seemed to be in a fast, concentration-dependent equilibrium with each other, which is in agreement with previous studies on IFNα-2b.^45^ Our experiments showed a clear correlation between pH and the formation of soluble oligomers. Our many-body simulations indicated a general trend toward larger average cluster size with increasing pH, which was in good agreement with experimental results. Analysis of the cluster shape in our simulations showed more spherical, less defined clusters when the cluster formation is increased. Our SAXS measurements, however, showed elongated oligomers. Even though we believe the main driving forces for IFNα-2a oligomerization and aggregation are electrostatic interactions, hydrophobic interactions, which are unaccounted for in our simulations, might influence the self-association of IFNα-2a.

Our experimental measurements in combination with the Monte Carlo simulations point toward different types of protein–protein interaction for IFNα-2a. The formation of large insoluble aggregates and protein crystals could be diminished by the presence of salt. The formation of smaller soluble oligomers appeared to be less influenced by the presence of salt but very much pH and protein concentration dependent. From a pharmaceutical point of view, irreversible aggregation can lead to compromised activity and immune response in patients. We found the least number of insoluble aggregates at low pH (pH 5) in the presence of salt (140 mM NaCl). This formulation resembles the commercial formulation of IFNα-2a (Roferon-A; 10 mM ammonium acetate buffer pH 5, 120 mM NaCl) closely. As IFNα-2a has been reported to be acid labile and undergoes partial unfolding at pH 4,^18^ this formulation likely represents the optimal conditions to prevent aggregation. However, other IFNα products (IFNα-2b (Intron-A), Interferon aphacon-1 (Infergen) and IFNα-n3 (Alferon)) are formulated in phosphate buffer at pH 7 despite studies that show self-association under this condition.^45,59^ This might be explained by the low protein concentration in commercial formulation, which shifts the equilibrium toward the monomeric form as we could show in our study.

IFNα-2a only formed protein crystals when no salt was present, indicating that the crystal interactions are most likely formed by ionic interactions. The crystal formation has not been reported in any previous studies of IFNα-2a.^16−20^ SAXS is currently the only technique applied for IFNα-2a characterization that can show the presence of protein crystals apart from visual inspection in a microscope. Remarkably, these IFNα-2a protein crystals formed without zinc(II), which raises the question of whether the zinc being a mediator, as reported for the dimerization of IFNα-2b and IFNβ,^50,60^ is necessary to form interferon crystals. The pH of IFNα-2b and IFNβ crystal formation is close to pH 5, indicating that some protein–protein interactions are similar within the group of interferons. Solving the crystal structure of IFNα-2a would highly benefit this study but has not been possible to date as the obtained crystals were too fragile to handle.

The measurement of the stability of IFNα-2a toward thermal and chemical denaturation showed a lower stability in the presence of salt at pH 5, which is in agreement with previous studies.^17−20^ The contrast between IFNα-2a conformational stability below and above its pI is remarkable. These measurements indicate, that in the case of a predominantly negatively charged protein, the presence of salt enhances IFNα-2a’s conformational stability, while it is lower in the case of a predominantly positively charged protein. This is in agreement with previous studies on IFNα-2b.^59^ The conformational stability of IFNα-2a was independent of the colloidal stability and shows that a combination of different variables in a screening for stability is important and cannot be generalized. The concentration of IFNα-2a had no influence on IFNα-2a’s colloidal and conformational stability, which was indicated in previous studies.^16^

## Conclusion

Through systematic biophysical and computational characterization of IFNα-2a, we gained molecular insights into the driving forces of the oligomerization and aggregation. With this combination of methods, we could assess the challenge of characterizing a polydisperse system as IFNα-2a in a new way. Because of the sequence and structural similarity among cytokines, our results benefit the study of these powerful protein drugs. Formulation and reformulation have no impact on the results, which could lead to new approaches to the formulation of protein drugs. They could, for example, be stored at low pH, a condition where they show high stability as indicated in our study, and reformulated just before treatment into conditions where they show lower stability but are more beneficial for the patient.

The data presented in this study will be connected with similar data sets on other types of protein drugs in the context of the EU international training network project on ‘protein excipient and protein–protein interaction in formulation (PIPPI)’.^62^ This will finally result in a comprehensive data set characterizing different kinds of protein drugs as a function of formulation conditions and facilitate a better understanding of the solution behavior of protein drugs.

## Supporting Information Available

Sequence and structural alignment within the Interferon class; additional SAXS parameters and analysis (full SAXS curves, *p*(*r*) functions, *ab initio* modeling); calculated biophysical parameters; additional DLS and ICD measurements in addition of excipients; quantitative AUC analysis; supplementary discussion regarding dimer assembly (PDF)

## Notes

Electronic notebooks for reproducing the presented simulations and analysis are available at https://github.com/mlund/SI-interferon-oligomerization
