# Supplementary material for: Electrostatics Drive Oligomerization and Aggregation of Human Interferon Alpha-2a
Source: J Phys Chem B. 2021 Dec 13;125(50):13657–69. doi: 10.1021/acs.jpcb.1c07090 (PMC8713289; doi:10.1021/acs.jpcb.1c07090)
Supplement: Supplementary file 1 — jp1c07090_si_001.pdf [file jp1c07090_si_001.pdf]

SUPPORTING INFORMATION

Electrostatics Drive Oligomerization and Aggregation of Human Interferon Alpha-2a

Christin Pohl[a,b,1,2]*, Marco Polimeni[c,1], Sowmya Indrakumar[b,3], Werner Streicher[a,4], Günther H.J. Peters[b], Allan Nørgaard[a], Mikael Lund[c,5,] and Pernille Harris[b,6]*

[a]Novozymes A/S, 2880 Bagsvaerd, Denmark
[b]Technical University of Denmark, Department of Chemistry, 2800 Kongens Lyngby, Denmark
[c]Lund University, Division of Theoretical Chemistry, Department of Chemistry, 221 00 Lund, Sweden
[1]these authors contributed equally
[2]present address: Lund University, Division of Biochemistry and Structural Biology, Department of Chemistry, 221 00 Lund, Sweden
[3]present address: Department of Biology, University of Copenhagen, 2100 Copenhagen, Denmark
[4]present address: NanoTemper Technologies GmbH, Muenchen, Germany
[5]secondary address: Lund Institute of advanced Neutron and X-ray Science (LINXS), 22370 Lund, Sweden
[6]present address: Department of Chemistry, University of Copenhagen, 2100 Copenhagen, Denmark
*corresponding authors: C.P.: christin.pohl@biochemistry.lu.se; P.H.: phharris@chem.ku.dk

Table S1: Sequence and structural identity of IFNα-2a (PDBID:1IFT), IFNα-2b (PDBID:1RH2), human IFNβ (PDBID: 1AU1) and murine IFNβ (PDBID:1WU3 26).

	Protein	Sequnce identity (%)	Structural identity RMSD (Å)
PDBID: 1ITF	IFNα-2a	100	0
PDBID: 1RH2	IFNα-2b	99.39	0.95
PDBID: 1AU1	murine IFNβ	34.57	1.602
PDBID: 1WU3	human IFNβ	30.38	1.786

Table S2: Clustal X Color scheme for multiple sequence alignment used for Figure S1. The residue is colored when the threshold on position x is reached within the given residue group.

Amino acid group	Color	Residue	Threshold in residue group
Hydrophobic	PETROL	A,I,L,M,F,W,V	>60% in WLVIMAFCHP
		C	>60% in WLVIMAFCHP
Positive charge	MAGENTA	K,R	>60% in KR or >80% in KRQ
Negative charge	RED	E	>60% in KR or >50% in QE or >85% in EQD
		D	>60% in KR or >85% in KRQ or >85% in ED
Polar	GREEN	N	>50% in N or >85% in NY
		Q	>60% in KR or >50% in QE or >85% in QEKR
		S,T	>60% in WLVIMAFCHP or >50% in TS or >85% in ST
Cysteines	PINK	C	>85% in C
Glycines	GREY	G	>0% in G
Prolines	YELLOW	P	>0% in P
Aromatic	CYAN	H,Y	>60% in WLVIMAFCHP or >85% in WYACPQFHILMV
Unconserved	WHITE	any/gap	if none of the above criteria are met

Table S3: Overview over concentrations and derived values of $I(0)$, R_g (nm) and apparent MW (kDa) from SAXS measurements.

Buffer	c (mg/ml)	Guinier analysis $I(0)/c$	Guinier analysis R_g (nm)	$p(r)$ analysis $I(0)/c$	$p(r)$ analysis R_g (nm)	Apparent MW (kDa) Guinier	Apparent MW (kDa) $p(r)$
10 mM His pH 5	1.0	Bragg peaks					
	2.0						
	5.0						
	7.0						
	10.0						
	15.0						
	20.0						
	30.0						
10 mM His pH 5 140 mM NaCl	0.7	0.023	2.41	0.022	2.34	31.9	30.5
	1.7	0.030	2.74	0.029	2.70	41.5	40.8
	2.6	0.036	3.03	0.035	2.99	49.9	49.1
	3.8	0.041	3.26	0.041	3.31	56.8	56.2
	4.5	0.043	3.41	0.043	3.45	59.6	59.8
	6.4	0.051	3.71	0.051	3.75	70.6	70.3
	6.9	0.057	4.05	0.058	4.44	78.9	80.3
10 mM His pH 7.5	1.1	0.066	4.06	0.065	4.02	91.4	89.9
	2.9	0.086	4.62	0.085	4.73	119.1	118.3
	5.4	0.095	4.64	0.099	5.10	131.6	136.8
	6.9	0.098	4.56	0.102	5.00	135.7	141.1
	9.4	0.096	4.20	0.107	5.08	133.0	148.2
	10.7	0.100	4.47	0.105	4.86	138.5	145.6
	11.4	0.100	4.57	0.108	4.94	138.5	149.9
	11.8	0.100	4.50	0.105	4.82	138.5	145.8
10 mM His pH 7.5 140 mM NaCl	1.2	0.058	4.11	0.058	4.25	80.3	80.0
	2.3	0.076	4.68	0.075	4.73	105.5	103.4
	5.2	0.120	7.07	0.106	5.75	166.2	146.3
	7.3	0.140	7.78	0.132	7.56	193.9	182.3
	10.9	0.140	7.63	0.147	8.33	193.9	202.9
	13.6	0.140	7.09	0.136	7.36	193.9	188.8
10 mM Ace pH 5	1.0	Bragg peaks					
	2.0						
	5.0						
	7.0						
	10.0						
	15.0						
	20.0						
	30.0						
10 mM Phos pH 7.5	1.0	0.100	4.30	0.102	4.46	138.5	141.3
	2.0	0.110	4.47	0.108	4.69	152.3	149.4
	5.0	0.150	5.29	0.155	6.03	207.7	214.0
	7.0	0.150	5.29	0.148	5.15	207.7	205.0
	10.0	0.140	5.75	0.138	4.92	193.9	191.3
	15.0	0.140	5.01	0.114	3.92	193.9	158.0
	20.0	0.120	4.39	0.122	4.50	166.2	169.1

Table S4: Theoretical biophysical parameters of different constructed oligomers of IFNα-2a based on the alignment to human IFNβ´s crystal structure (PDBID:1AU1) calculated using HULLRAD.

**biophysical parameters**	**INFα-2a**
MW (Da)	19244
v-bar (ml/g)	0.739
R_g (Å)	15.86
D_{max} (Å)	50.07
f/f0	1.20
sed. coeff. (s)	2.08
R_h (Å)	22.71

Table S5: Quantitative analysis of IFNα-2a oligomers present at different protein concentrations and conditions in AUC measurements. Parameters were determined from the maximum peak position and the integrated peak area.

		peak 1			peak 2			peak 3		
		c =0.2 (mg/ml)	*c* =0.4 (mg/ml)	*c* =0.8 (mg/ml)	*c* =0.2 (mg/ml)	*c* =0.4 (mg/ml)	*c* =0.8 (mg/ml)	*c* =0.2 (mg/ml)	*c* =0.4 (mg/ml)	*c* =0.8 (mg/ml)
**pH 5**	sed. coeff. (s)	2.28	2.36	2.43	~3.20	3.25	3.47	not consistent/small fraction		
	percent	95.3	57.56	45.43	4.55	32.44	48.62			
**pH 5, 140 mM NaCl**	sed. coeff. (s)	2.21	2.21	2.21	3.47	3.1	3.03	not consistent/small fraction		
	percent	87.74	90.23	69.98	6.7	8.32	29.04			
**pH 7.5**	sed. coeff. (s)	~2.40	~2.80	2.06	3.99	4.29	4.51	4.96	5.48	5.7
	percent	3.78	1.24	0.7	24.06	22.57	13.12	63.35	71.66	82.09
**pH 7.5, 140 mM NaCl**	sed. coeff. (s)	~2.00	1.94	2.04	3.03	3.25	3.32	4.07	4.66	5.26
	percent	4.48	2.37	1.16	45.17	32.12	16.29	41.83	63.62	78.5

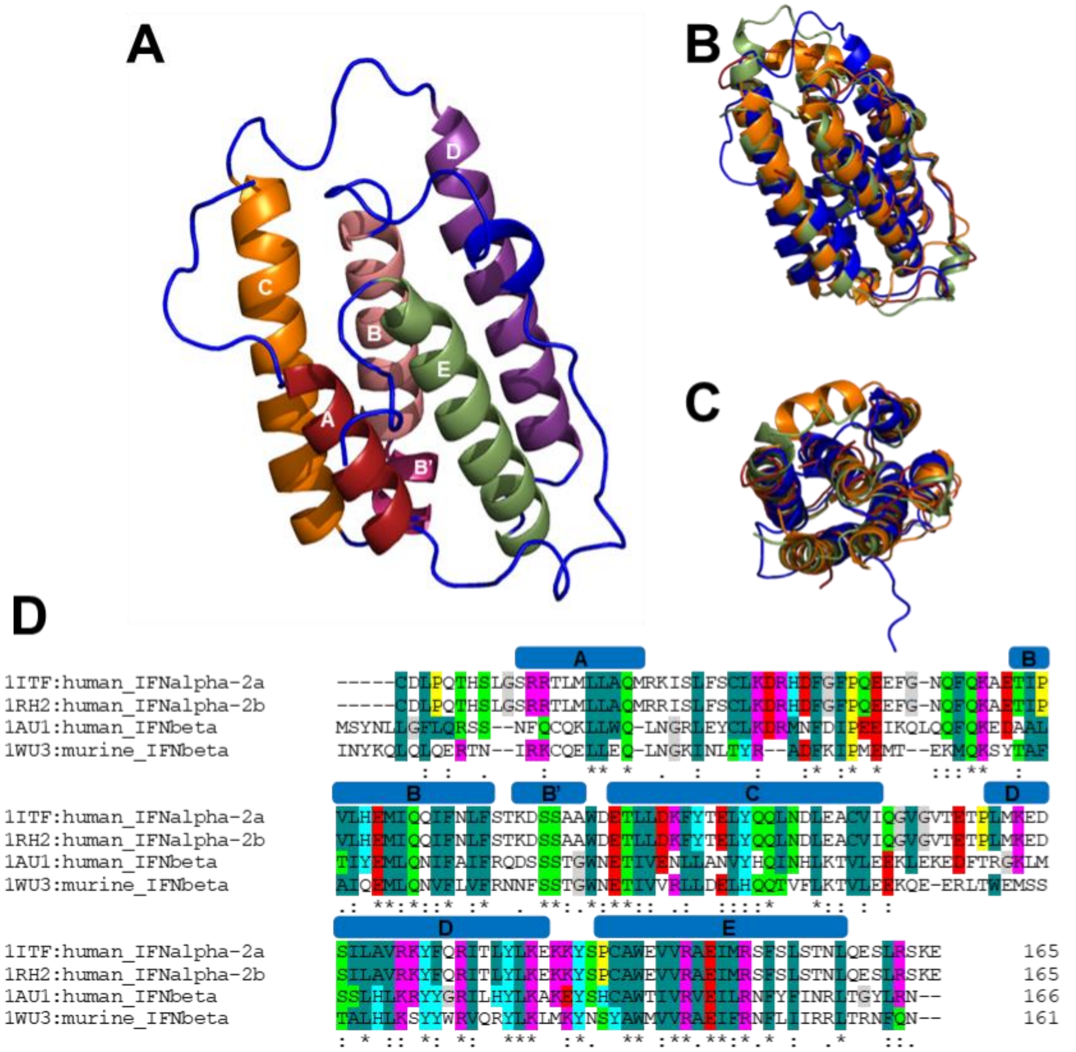

Figure S1: A: IFNα-2a high-resolution structure (PDBID: 1ITF[1]). B, C: structural alignment of IFNα-2a (blue), IFNα-2b (PDB ID:1RH2[2])(red) , human IFNβ (PDB ID: 1AU1[3])(green) and murine IFNβ (PDB ID:1WU3[4]) (orange). C shows alignment 90º rotated compared to B. Figures were made using PyMOL[5]. D: Sequence alignment with CLUSTAL OMEGA (version 1.2.4) using the CLUSTAL X color scheme (Table S2).

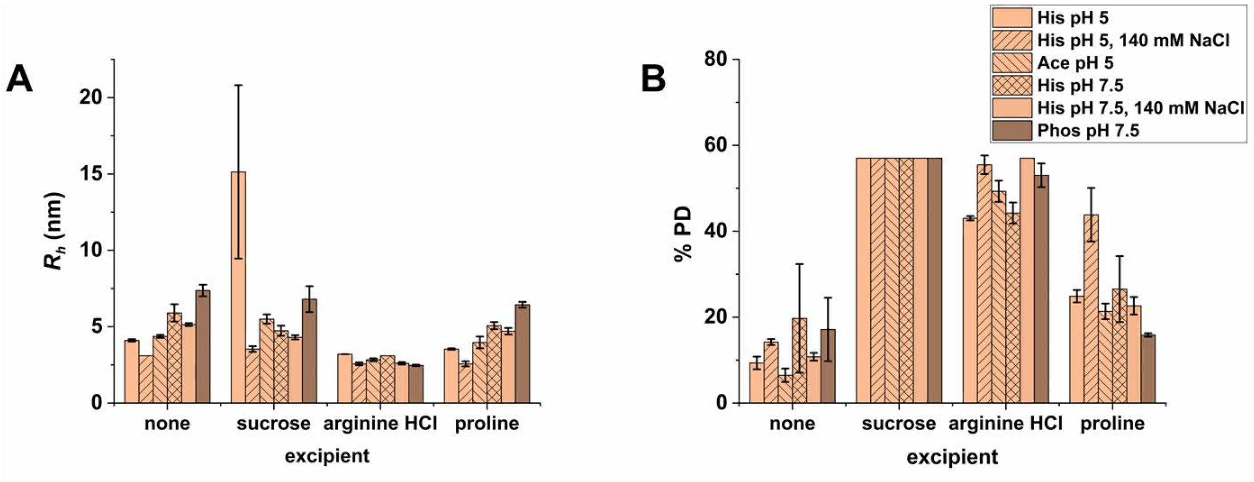

Figure S2: Colloidal stability of IFNα-2a in addition of the excipients sucrose (280 mM), arginine HCl (140 mM) and proline (280 mM) and in different buffer systems. A: Apparent Rh (nm). B: percent polydispersity %PD. Data is mean ± S.D. for 3 replicates.

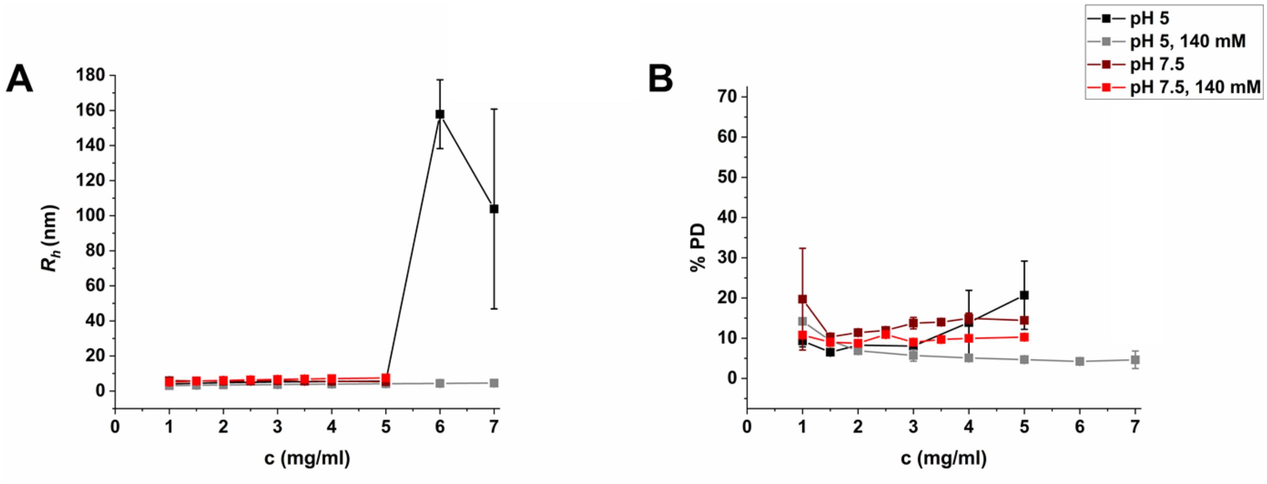

Figure S3: Colloidal stability of INFα-2a at pH 5 and pH 7.5 with and without salt as a function of protein concentration measured with DLS. A: Hydrodynamic radius R_h (nm). B: percent polydispersity %PD. Data is mean ± S.D. for 3 replicates.

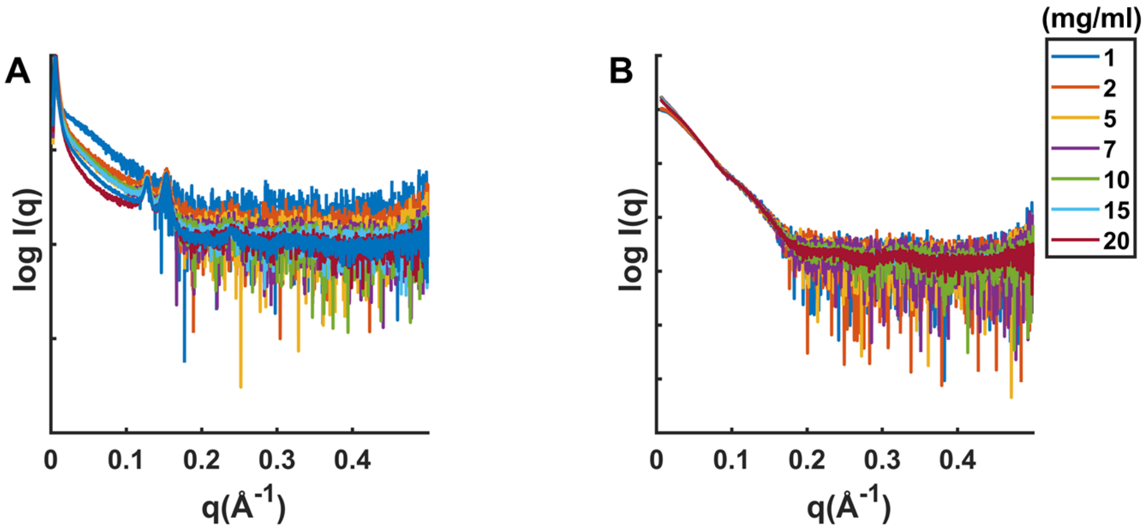

Figure S4: SAXS curves of IFNα-2a in different buffers. A: 10 mM acetate pH 5. B: 10 mM phosphate pH 7.5.

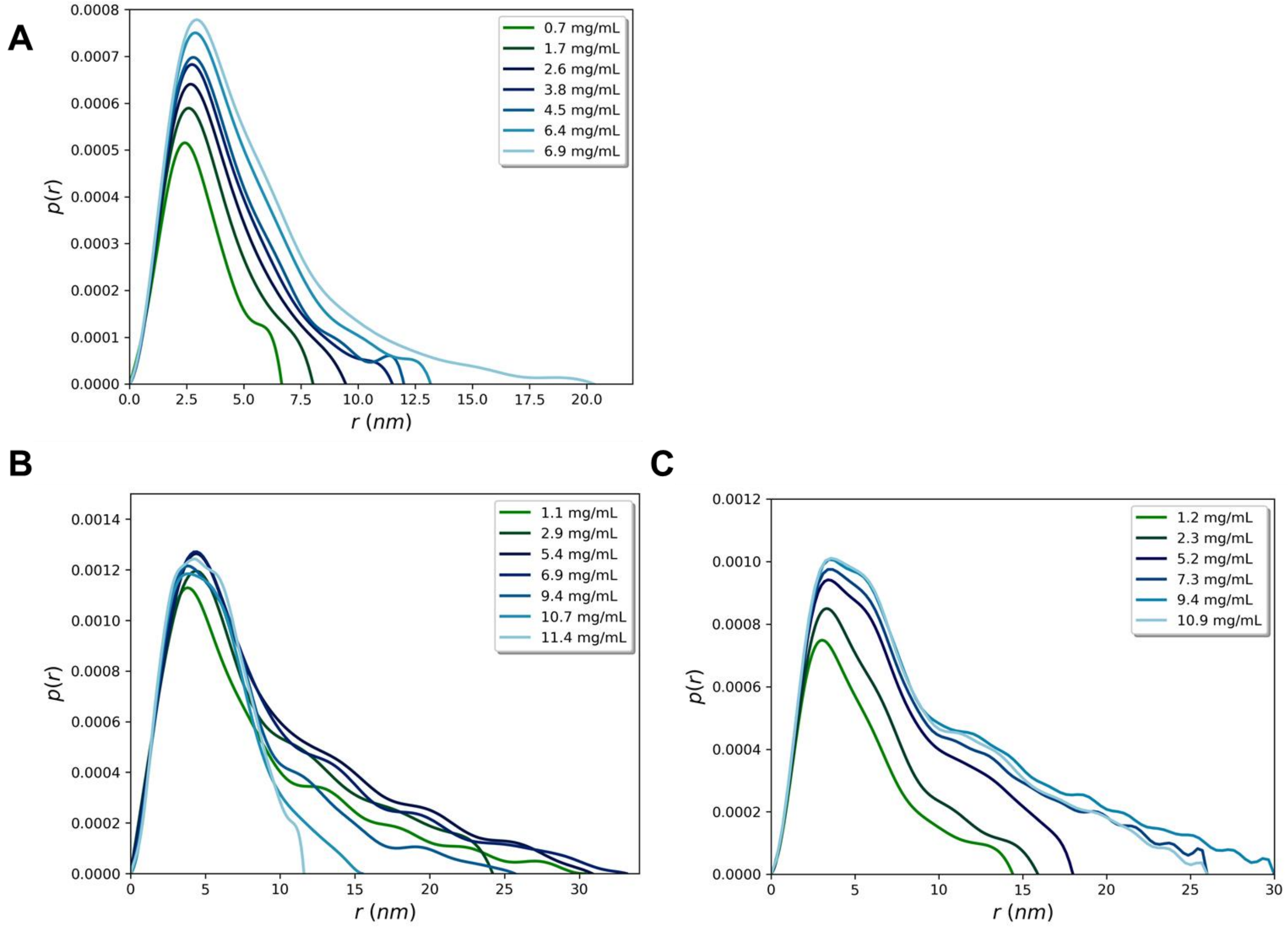

Figure S5: SAXS measurements of IFNα-2a at different concentrations presented as distance distribution *p*(*r*) function. A: Histidine pH 5, 140 mM NaCl. B: Histidine pH 7.5. C: Histidine pH 7.5, 140 mM NaCl.

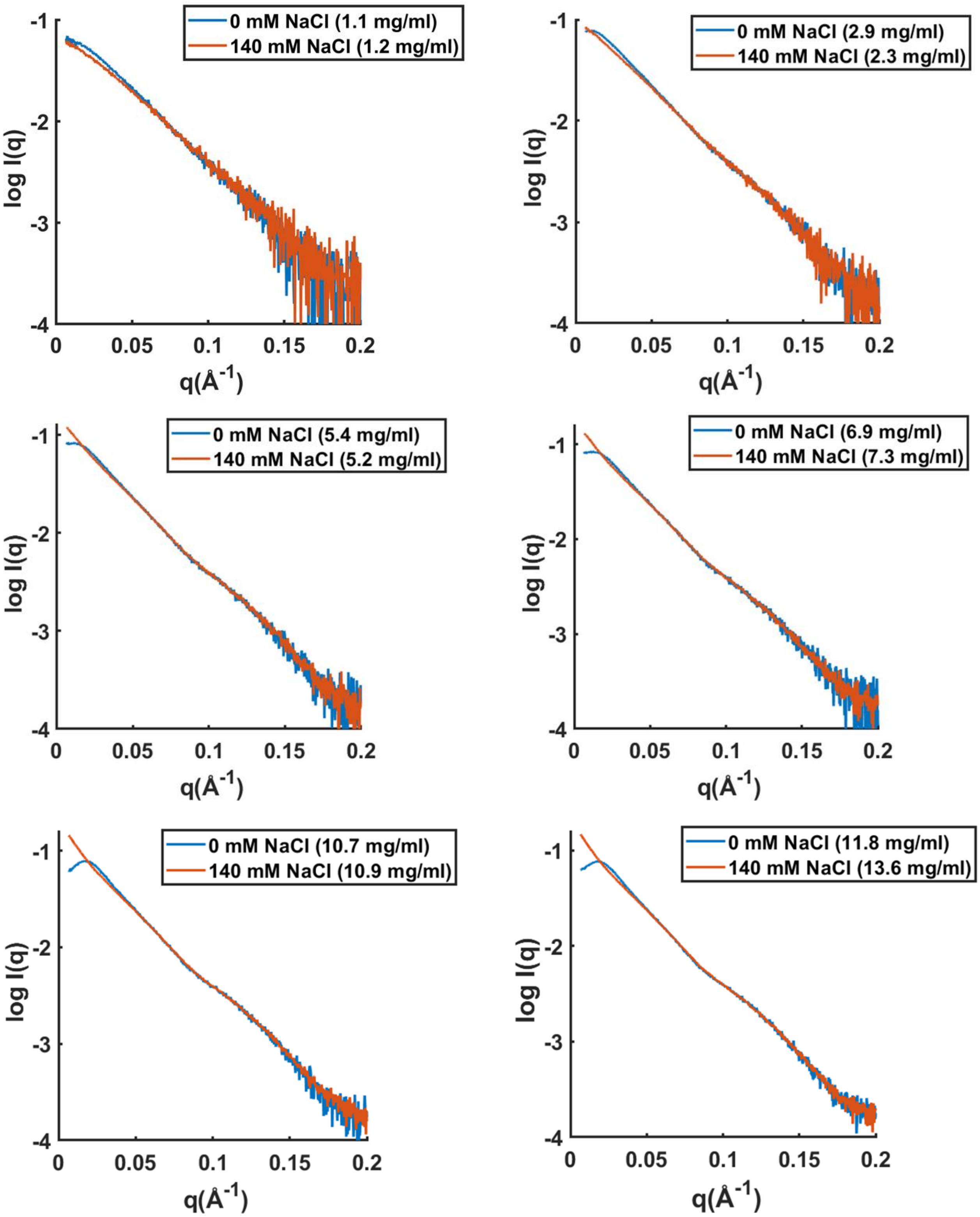

Figure S6: Direct comparison of SAXS curves of IFNα-2a in histidine pH 7.5 at different protein concentrations in with salt (red) and without salt (blue).

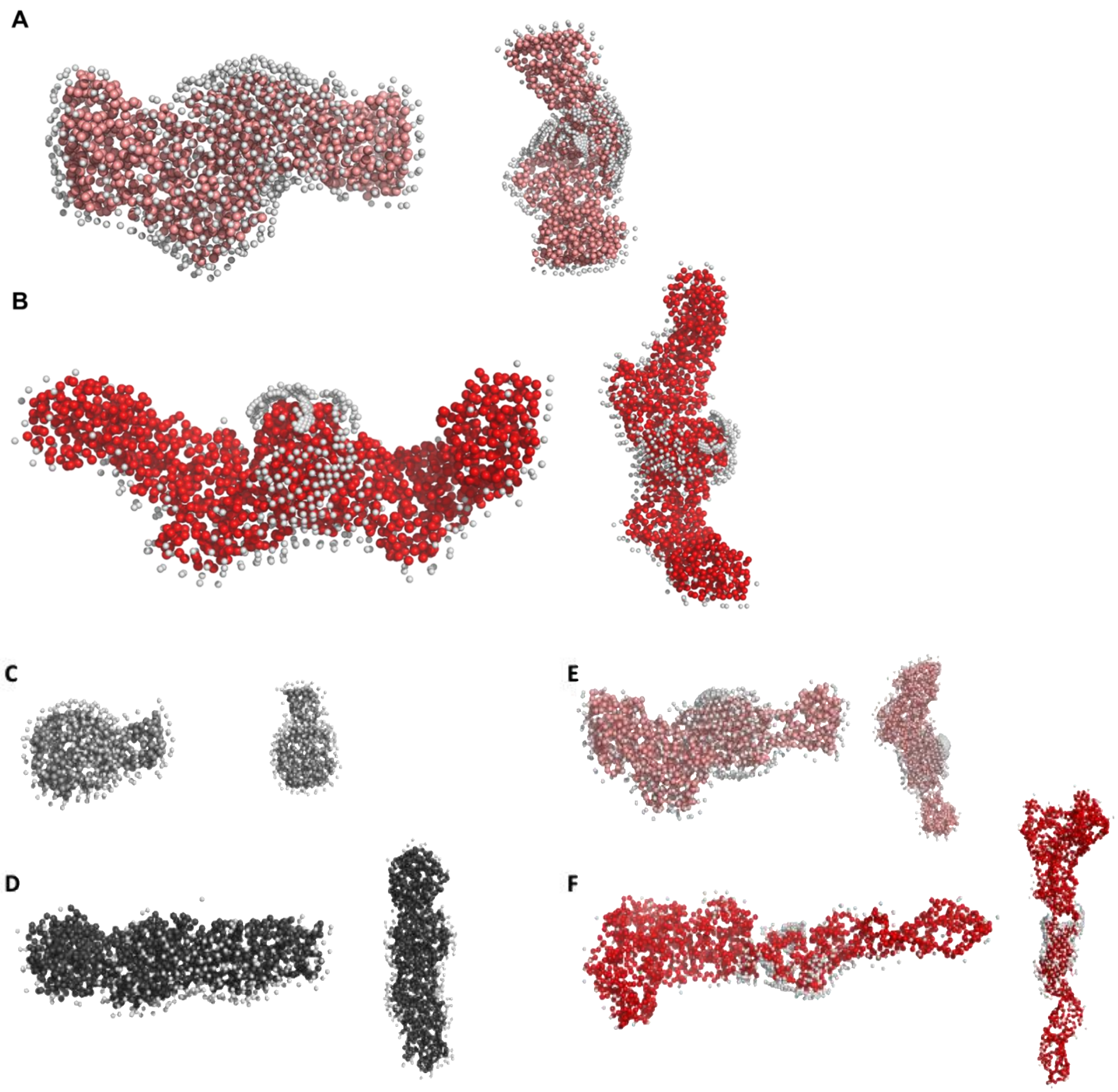

Figure S7: *Ab-initio* models of IFNα-2a (program: GASBOR). *Ab-initio* models are shown as spheres with one bead representing one C_α atom. A: *Ab-initio* model at pH 7.5, *c*=1 mg/ml (χ^2=0.69). B: *Ab-initio* model at pH 7.5, *c*=7 mg/ml (χ^2= 0.43). C: *Ab-initio* model at pH 5, 140 mM NaCl, *c*=1 mg/ml (χ^2=0.61). D: *Ab-initio* model at pH 5, 140 mM NaCl, *c*=7 mg/ml (χ^2=1.2). E: *Ab-initio* model at pH 7.5, 140 mM NaCl, *c*=1 mg/ml (χ^2=0.83). F: *Ab-initio* model at pH 7.5, 140 mM NaCl, *c*=7 mg/ml, (χ^2= 0.59).

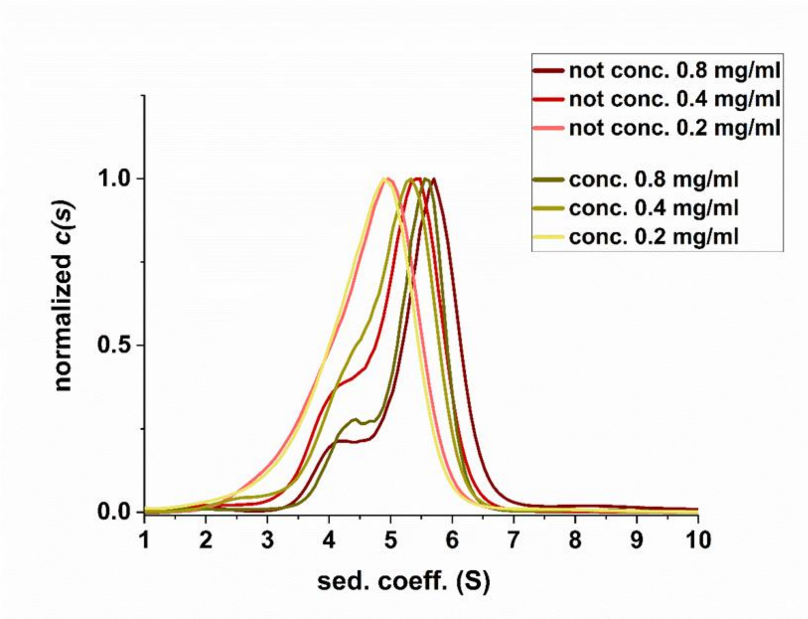

Figure S8: Comparison of a non-concentrated sample (red) and a concentrated sample prior to dialysis (yellow) of IFNα-2a oligomers of formulated samples in histidine pH 7.5 measurement with AUC.

SUPPORTING DISCUSSION

In order to investigate how IFNα-2a structurally assembled into soluble oligomers and insoluble aggregates and protein crystals, we tried to find a model which fits our experimental data best. The *ab-initio* modelling of the SAXS data showed an elongated, screw-shaped average for the oligomers formed. This modelling is based on a polydisperse system, and it is important to be aware that in this case it does not represent only one oligomer formed, but an average shape of the oligomers formed by IFNα-2a. The shape of the model appeared to be independent of the presence of salt. As IFNα-2a was not monodisperse in solution, fitting the data with a model and drawing conclusions about the assembly of the oligomeric species is challenging. To simplify the system, we tried to draw conclusions about the assembly of the IFNα-2a dimer, the smallest oligomer a protein can form, by combining our experimental data with computational approaches. IFN dimers in the published crystal structures of human IFNβ (PBD ID: 1AU1) and human IFNα-2b (PDB ID:1RH2) showed, despite their high structural identity (Figure S1), different orientations of the two monomers. Based on the crystal contacts in these two structures, we constructed three possible dimers of different orientations by alignment. Dimer 1 represents the alignment to human IFNβ dimer and dimer 2, the alignment to human IFNα-2b. Dimer 3 represents the alignment to the perpendicular interface, which showed similar crystal contacts in both structures. The theoretical SAXS curves (CRYSOL) of the constructed dimers were fitted against the experimental data of IFNα-2a at pH 7.5 (Figure S9). The comparison of the theoretical SAXS curve of constructed possible dimers with the experimental data showed significant differences for all constructed dimers, which was due to the presence of different oligomers in the sample. However, the curve shape of the parallel-oriented dimer followed the middle part of the SAXS curve the best (Figure S9). Additionally, we analyzed the charge distribution of all constructed dimers as a function of pH and salt concentration (Figure S10). A change of pH and salt led only to minor changes in the electrostatics. For dimer 2 and dimer 3, we observed strong repulsive electrostatics in the dimer interface. For dimer 1, we observed negative and positive electrostatics in the dimer interface. We believe therefore, that IFNα-2a might assemble in a similar orientation as dimer 1. However, different types of interactions most likely play a role in the protein-protein interactions leading to oligomerization and aggregation of IFNα-2a and it is difficult to say whether the dimerization of IFNα-2a modeled based on soluble oligomer in our SAXS experiments are comparable with crystal contacts.

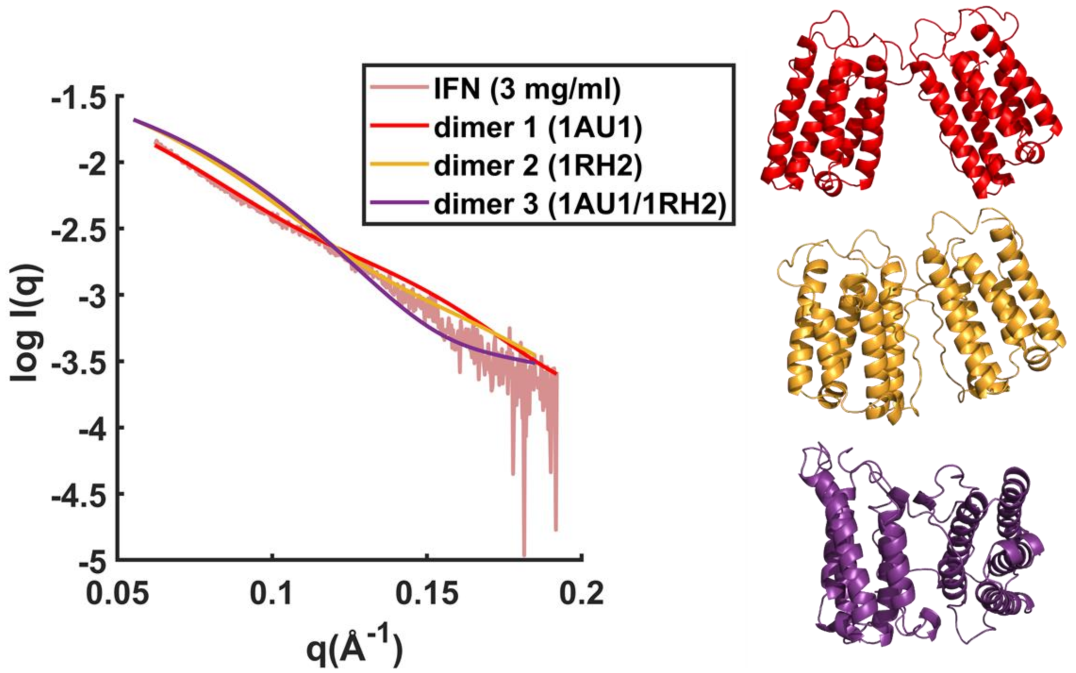

Figure S9: Theoretical SAXS curves of different dimers of IFNα-2a constructed based on alignment to published crystal structures of structurally similar interferons using PyMOL[5]. The IFNα-2a monomer was aligned to human IFNβ (PBD: 1AU1[3]) (dimer 1, red) and human IFNα-2b (PDB:1RH2[2]) (dimer 2, yellow) and the interface of perpendicular oriented α-helices, which was similar in both crystal structures (dimer 3, purple). The theoretical SAXS curve of the constructed dimers was created using CRYSOL[6] and compared to the experimental curve of IFNα-2a at pH 7.5.

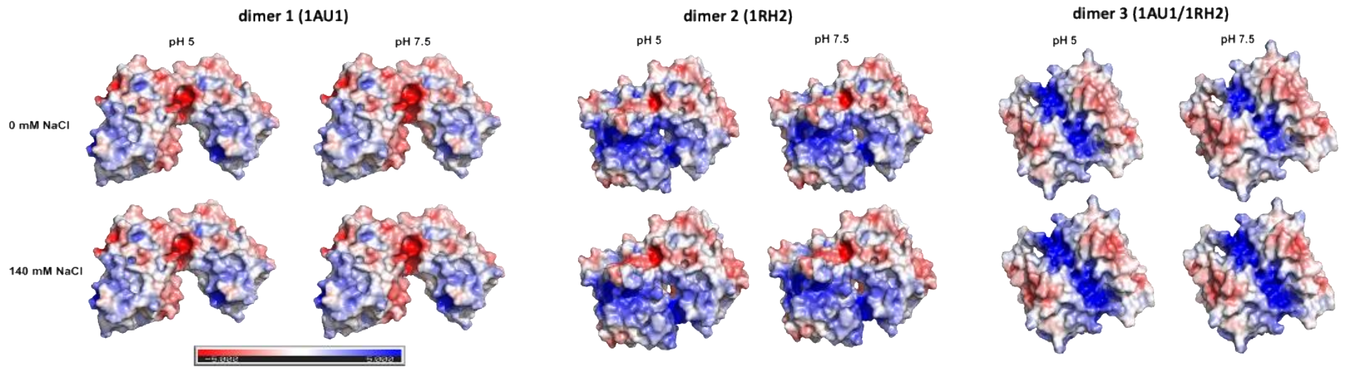

Figure S10: Electrostatic properties of constructed dimers of IFNα-2a computed by H++[7–9]. Dimer 1: IFNα-2a monomer (PDB ID: 1ITF[1]) was aligned to the published crystal structure of human IFNβ (PBD ID: 1AU1[3]) and human IFNα-2b (PDB ID: 1RH2[2]) using PyMOL. Negative electrostatics are indicated in red, positive electrostatics are indicated in blue. Dimer 1: alignment to human IFNβ. Dimer 2: alignment to human IFNα-2b. Dimer 3: alignment to perpendicular interface in human IFNβ and IFNα-2b.

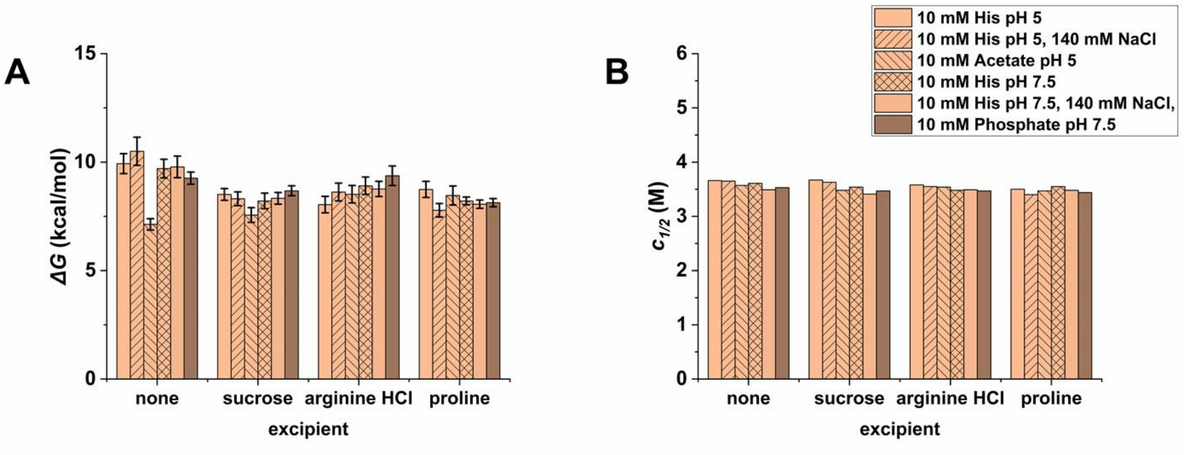

Figure S11: Conformational stability of IFNα-2a in addition of the excipients sucrose (280 mM), arginine•HCl (140 mM) and proline (280 mM) and in different buffer systems. A: Gibbs free energy of unfolding Δ*G* by chemical denaturation. B: Inflection point $c_{1/2}$ of unfolding by chemical denaturation.

REFERENCES

1. Klaus, W., Gsell, B., Labhardt, A. M., Wipf, B. & Senn, H. The three-dimensional high resolution structure of human interferon α-2a determined by heteronuclear NMR spectroscopy in solution. *J. Mol. Biol.* **274**, 661–675 (1997).
2. Radhakrishnan, R. *et al.* Zinc mediated dimer of human interferon-α(2b) revealed by X-ray crystallography. *Structure* **4**, 1453–1463 (1996).
3. Karpusas, M. *et al.* The crystal structure of human interferon β at 2.2-Å resolution. *Proc. Natl. Acad. Sci. U. S. A.* **94**, 11813–11818 (1997).
4. Senda, T., Saitoh, S. I. & Mitsui, Y. Refined crystal structure of recombinant murine interferon-β at 2.15 Å resolution. *J. Mol. Biol.* **253**, 187–207 (1995).
5. Schrödinger, LLC. *The {PyMOL} Molecular Graphics System, Version~1.8*. (2015).
6. Svergun, D., Barberato, C. & Koch, M. H. CRYSOL - A program to evaluate X-ray solution scattering of biological macromolecules from atomic coordinates. *J. Appl. Crystallogr.* **28**, 768–773 (1995).
7. Anandakrishnan, R., Aguilar, B. & Onufriev, A. V. H++ 3.0: Automating pK prediction and the preparation of biomolecular structures for atomistic molecular modeling and simulations. *Nucleic Acids Res.* **40**, 537–541 (2012).
8. Myers, J., Grothaus, G., Narayanan, S. & Onufriev, A. A simple clustering algorithm can be accurate enough for use in calculations of pKs in macromolecules. *Proteins* **63**, 928–938 (2006).
9. Gordon, J. C. *et al.* H++: A server for estimating pKas and adding missing hydrogens to macromolecules. *Nucleic Acids Res.* **33**, 368–371 (2005).
